# Changes in frailty and incident risk of degenerative bone and joint diseases and their multimorbidity: a prospective cohort study

**DOI:** 10.3389/fpubh.2026.1831566

**Published:** 2026-05-08

**Authors:** Minghao Jin, Jiawei Liu, Xin Song, Bowen Lei, Yan Su, Zhongyuan Shi, Dan Xu, Bin Yang, Di Zhang, Xiaofeng Ma, Hao Li, Zilan Chen, Zihao Li, Yuqi Pang, Yangdan Zhong, Maoyao Xia, Ye Ju, Zhi Feng, Mengyu Fan, Xia Jiang

**Affiliations:** 1 Department of Epidemiology and Biostatistics, West China School of Public Health and West China Fourth Hospital, Sichuan University, Chengdu, Sichuan, China; 2 Department of Nutrition and Food Hygiene, West China School of Public Health and West China Fourth Hospital, Sichuan University, Chengdu, China; 3 Department of Clinical Neuroscience, Karolinska Institute, Stockholm, Sweden

**Keywords:** cohort study, degenerative bone and joint diseases, dynamic nature, frailty, reversibility

## Abstract

**Background:**

Frailty reflects multisystem physiological vulnerability and has been associated with an array of adverse health outcomes. However, evidence on how frailty and its longitudinal changes relate to degenerative bone and joint diseases (DBJDs) and their multimorbidity remains limited.

**Methods:**

We conducted a prospective cohort study using data from the UK Biobank (UKB). Frailty was evaluated using a validated frailty index (FI). Changes in frailty were characterized by frailty status transitions, the rate of change in FI (ΔFI), and cumulative burden (total FI). Incident DBJDs including osteoporosis, osteoarthritis, and intervertebral disc degeneration as well as degenerative bone and joint multimorbidity (DBJM) were ascertained through linkage to health records. Cox regression models were used to estimate the hazard ratios (HRs) and 95% confidence intervals (95%CIs).

**Results:**

Compared with baseline non-frail participants, frail individuals had substantially higher risks of both DBJDs (HR = 2.06, 95%CI = 2.01–2.11) and DBJM (HR = 3.89, 95%CI = 3.62–4.18), with pre-frail participants showing intermediate risks. In transitions analyses, compared with participants who remained stable, those who progressed to worse status had increased risks of DBJDs (non-frail → pre-frail/frail: HR = 1.33, 95%CI = 1.21–1.47; pre-frail → frail: HR = 1.39, 95%CI = 1.20–1.60). In contrast, frailty recovery from pre-frailty to non-frailty was associated with a decreased risk of DBJDs (HR = 0.80, 95%CI = 0.71–0.91), although evidence for frailty recovery was less consistent overall.

**Conclusion:**

Frailty status and its longitudinal changes are strongly associated with the risk of incident DBJDs and DBJM. Frailty progression and cumulative frailty burden confer substantially increased risks, while frailty recovery may be associated with a lower risk, although the evidence remains limited.

## 1 Introduction

Degenerative bone and joint diseases (DBJDs), including osteoporosis (OP), osteoarthritis (OA), and intervertebral disc degeneration (IVDD), are highly prevalent chronic musculoskeletal conditions characterized by progressive structural and functional deteriorations of bones and joints (1–6). These disorders have strong age-dependent etiology and constitute leading causes of chronic pain, limited mobility, and reduced quality of life among the middle-aged and older adults (7, 8). Beyond a high prevalence attributable to rapid global aging, DBJDs frequently co-occur within individuals as degenerative bone and joint multimorbidity (DBJM), leading to substantially greater public health and clinical burden (9). Given the insidious and progressive nature of DBJDs and their multimorbidity, identifying integrative, dynamic indicators that can capture early vulnerability and predict long-term risk of DBJDs and their accumulation becomes crucial, particularly during midlife and early older age when preventive strategies may still yield benefits.

Frailty is a multidimensional age-related condition characterized by reduced physiological reserve across multiple organ systems and increased vulnerability to external stressors (10–15). As an integrative measure of biological aging, frailty has been increasingly recognized as a powerful predictor of adverse health outcomes and a useful framework for risk stratification and targeted intervention, showing strong associations with a wide range of chronic diseases, including cardiovascular disease, type 2 diabetes, and cognitive impairment (16–18). Given the close interconnections among musculoskeletal function, physical activity, inflammation, and systemic aging processes, frailty may play an important role in the development and progression of DBJDs.

Previous studies linking frailty to OP, OA, and IVDD suggest its potential role in increasing susceptibility to these conditions (19–29). Specifically, Di et al., found that frailty was longitudinally associated with an elevated risk of OP (HR = 2.11, 95%CI = 1.98–2.25) (19). Regarding OA, a 9-year follow-up study of 4,573 participants reported that higher baseline frailty was associated with incident OA (OR = 1.73, 95%CI = 1.52–1.97) (27). Beyond disease onset, frailty has also been shown to modify disease expression; in a cohort study of 3,271 participants, frailty moderated the association between radiographic knee OA severity and pain (28). With regard to IVDD, a systematic review of 29 studies indicates that frailty is associated with adverse outcomes in degenerative spine disease, including higher risks of mortality, major complications, and prolonged hospitalization (29). However, most prior studies have focused on frailty status assessed at single time point and examined its association with single musculoskeletal outcome. Few investigations have considered DBJDs as a combined phenotype, and even fewer have addressed how frailty relates to the accumulation of multiple degenerative bone and joint conditions.

Frailty has been increasingly recognized as a dynamic and reversible state, with individuals experiencing progression or recovery over time. Longitudinal studies have shown that changes in frailty are strongly associated with subsequent risks of mortality (30, 31), cardiovascular disease (32), metabolic disorders (33), depressive symptoms (34), and kidney function decline (35). Nevertheless, whether changes in frailty over time influence the risk of incident DBJDs and their multimorbidity remains largely unknown. Understanding their longitudinal relationship is important not only for elucidating underlying biological mechanisms but also for supporting frailty as a potentially modifiable target for the prevention of DBJDs and their multimorbidity in nowadays aging populations.

To address these knowledge gaps, we conducted a large-scale prospective cohort study using data from the UK Biobank (UKB). We aimed to examine the associations of baseline frailty status and long-term frailty changes with the risk of incident DBJDs, including OP, OA, and IVDD, as well as their multimorbidity (DBJM). We hypothesized that progression of frailty would be associated with an increased risk of DBJDs and DBJM, whereas improvement in frailty status would be associated with a reduced risk.

## 2 Methods

### 2.1 Study design and population

The UKB is a population-based prospective cohort that recruited over 500,000 participants from 22 assessment centers across the United Kingdom from 2006 to 2010. UKB received ethical approval from the North West Multi-Centre Research Ethics Committee (REC reference 21/NW/0157). All participants provided written informed consent at enrollment. Detailed information on the study design has been described elsewhere (36). Three follow-up assessments were initiated in 2012–2013, 2014, and 2019, respectively. Owing to a limited number of participants who completed the third assessment and its relatively short follow-up duration, data from this wave were not included in the present study. Consequently, data from baseline and the first or second follow-up assessment were used to evaluate changes in frailty status over time. For participants with data available from both follow-ups, data of the earlier visit were used.

**Figure 1:**
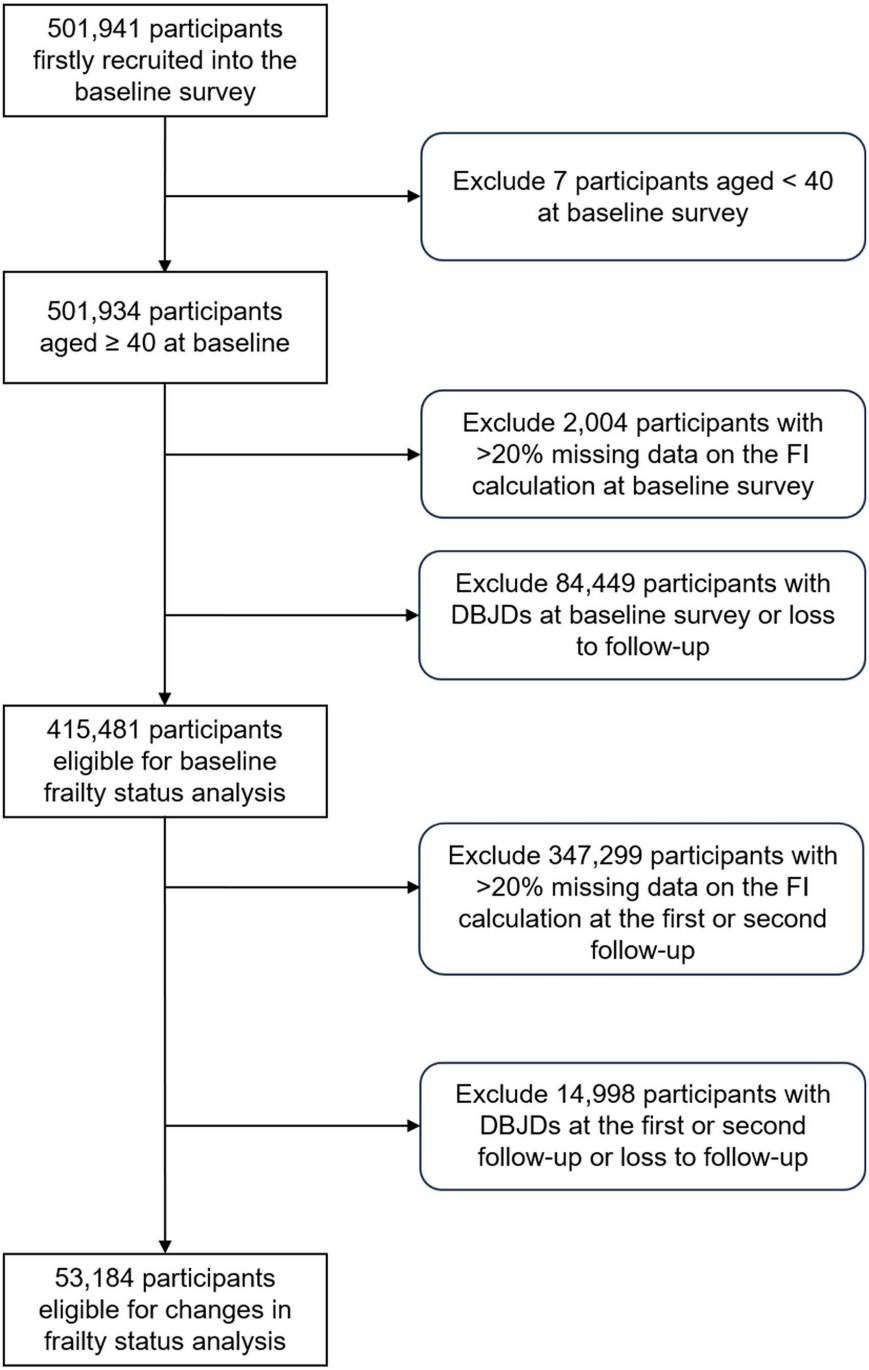
Selection process of the study population. FI, frailty index; DBJDs, degenerative bone and joint diseases.

Figure 1 illustrates the selection process for the study population. Participants were excluded if they were aged <40 years, had >20% missing data for baseline frailty index (FI) construction, had prevalent DBJDs at baseline, or were lost to follow-up (37). Consequently, 415,481 participants were included in the baseline frailty status analysis. For the analysis of changes in frailty status, we further excluded participants with >20% missing data to calculate FI at the first or second follow-up, as well as those who had DBJDs at the first or second follow-up or were lost to follow-up. Ultimately, 53,184 participants were included in the analysis of changes in frailty status.

### 2.2 Assessment of frailty

Frailty was assessed using the FI, which reflects the accumulation of multiple age-related health deficits (11, 38). In this study, the FI was calculated based on 36 self-reported questionnaire items related to diseases, symptoms, disabilities, physical function, cognition, and depression (Supplementary Table S1), following established methods for constructing the FI in UKB (39), with the exclusion of osteoporosis, osteoarthritis, rheumatoid arthritis, gout, and all pain-related items. Categorical variables were dichotomized (no deficit = 0; deficit = 1), while ordinal variables were rescaled to values between 0 and 1. For each participant, the FI was calculated as the sum of present deficits divided by 36, resulting in a continuous variable ranging from 0 to 1, with higher values indicating greater frailty. Consistent with previous studies, frailty status was categorized as: non-frail (FI ≤ 0.10), pre-frail (0.10 < FI ≤ 0.25), or frail (FI > 0.25) (32, 35, 46). Changes in frailty status were determined by comparing frailty status at baseline with those at the first or second follow-up. In addition to changes in frailty status, we also characterized the dynamic nature of frailty through two values ΔFI and total FI. ΔFI was calculated as the FI at the first or second follow-up minus the FI at baseline, while total FI was defined as the sum of the FI at baseline and the first or second follow-up.

### 2.3 Assessment of degenerative bone and joint diseases

The primary outcomes were DBJDs and DBJM. Cases were ascertained through International Classification of Diseases, Tenth Revision (ICD-10) diagnostic codes, including M80-M82 for OP, M15-M19 for OA, and G55.1, M51 for IVDD. Participants with a recorded diagnosis of OP, OA, or IVDD were considered to have DBJDs. DBJM was defined as the coexistence of two or three DBJDs in the same participant.

### 2.4 Assessment of covariates

Covariates included age, sex (female, male), race (white or other ethnic groups), education (less than or above the college/university degree), Townsend deprivation index (TDI; categorized into tertiles: T1, T2, and T3), smoking status (never, previous, current), drinking status (never, previous, current), physical activity level based on the International Physical Activity Questionnaire (IPAQ; low/moderate/high), and body mass index (BMI).

### 2.5 Statistical analyses

Descriptive statistics were summarized by frailty status at baseline. Continuous variables are presented as means (standard deviation, SD) or medians (interquartile range, IQR), and categorical variables as numbers (percentages). The missing data of covariates were imputed using multiple imputation by chained equations.

Cox proportional hazards models were used to estimate hazard ratios (HRs) and 95% confidence intervals (95%CI) for the association between baseline frailty status and the risk of incident DBJDs and DBJM. The proportional hazards assumption for all Cox regression models was verified using Schoenfeld residuals. Person-years of follow-up were calculated from the date of the baseline to the date of the first diagnosis of outcome, death, or the censoring date, whichever came first. Two models were fitted. Model 1 was unadjusted. Model 2 was multivariable-adjusted for age, sex, race, education, TDI, smoking status, drinking status, physical activity level and BMI. Using the same analytical framework, we further analyzed the associations of changes in frailty status, the rate of ΔFI, and total FI with incident DBJDs and DBJM. The study period was divided into two phases: person-years of interval were calculated from the date of baseline assessment to the date of the first or second follow-up. Person-years of follow-up were calculated from the date of the first or second follow-up to the date of the first diagnosis of outcome, death, or the censoring date, whichever came first. Both the rate of ΔFI and total FI were categorized into tertiles, with the lowest tertile serving as the reference. For analyses of the rate of ΔFI, Model 3 was additionally adjusted for baseline FI on top of the multivariable-adjusted model 2. Trend tests were conducted by modeling the rate of ΔFI and total FI as continuous variables. We further explored the associations of each 0.01-unit and 0.05-unit increment in these indices.

Stratified analyses were conducted by sex and age (middle-aged <60 years; older ≥60 years). The statistical significance of interaction terms was evaluated using the Wald test. Several sensitivity analyses were performed to test the robustness of primary findings. First, analyses were repeated using two alternative sets of FI cut-offs for defining frailty status: non-frail (FI ≤ 0.08), pre-frail (0.08 < FI ≤ 0.25), and frail (FI > 0.25); and non-frail (FI ≤ 0.10), pre-frail (0.10 < FI ≤ 0.21), and frail (FI > 0.21) (32, 33, 35). Second, to address potential reverse causality, we conducted sensitivity analyses excluded cases occurring within the first one or 2 years of follow-up.

All statistical analyses were performed using R software (version 4.5.1). All tests were two-sided, and a *p* value < 0.05 was considered statistically significant.

## 3 Results

### 3.1 Baseline characteristics of the study population

Among the 415,481 participants included in the baseline frailty status analysis (53.0% female, mean age 55.8 years), 43.4, 50.7, and 5.9% were classified as non-frail, pre-frail and frail status. Baseline characteristics according to frailty status are presented in Supplementary Table S2. Compared with non-frail participants, those who were pre-frail or frail were older, more likely to be female, less educated, of lower socioeconomic position, and less physically active. For the analysis of changes in frailty status, 53,184 participants were included (49.6% female, mean age 54.7 years), 52.0, 45.1 and 2.9% of the participants were classified as non-frail, pre-frail and frail status at baseline, with characteristics presented in Supplementary Table S3. The median interval time from the date of baseline assessment to the date of the first or second follow-up was 8.37 years.

### 3.2 Baseline frailty status and incident DBJDs and their multimorbidity

During a median follow-up of 13.31 years, 79,485 participants developed DBJDs and 8,809 participants developed DBJM. Baseline frailty status was strongly associated with the risk of incident DBJDs and DBJM (Table 1). After multivariate adjustment, compared with non-frail participants, frail participants had a significantly increased risk of DBJDs (HR = 2.06, 95%CI = 2.01–2.11). Similar associations were observed for individual disease outcomes (OP: HR = 3.14, 95%CI = 2.96–3.34; OA: HR = 1.90, 95%CI = 1.84–1.95; IVDD: HR = 2.82, 95%CI = 2.63–3.01). The association became particularly pronounced for DBJM, where frail participants had more than a threefold increased risk compared with non-frail participants (HR = 3.89, 95%CI = 3.62–4.18). Pre-frail participants also exhibited significantly elevated risks across all outcomes.

**Table 1:** Association of baseline frailty status with the risk of incident degenerative bone and joint diseases and their multimorbidity.

Variables	Cases/PYs	HR_1_ (95%CI)^a^	P_1_^a^	HR_2_ (95%CI)^b^	P_2_^b^
Degenerative bone and joint diseases					
Non-frail	25,864/2,252,245	Reference		Reference	
Pre-frail	45,175/2,496,461	1.58 (1.56, 1.61)	<0.001	1.36 (1.34, 1.38)	<0.001
Frail	8,446/265,275	2.82 (2.75, 2.89)	<0.001	2.06 (2.01, 2.11)	<0.001
Per 0.01-point increase		1.04 (1.04, 1.04)	<0.001	1.03 (1.03, 1.03)	<0.001
Per 0.05-point increase		1.24 (1.23, 1.24)	<0.001	1.17 (1.16, 1.17)	<0.001
Osteoporosis					
Non-frail	3,910/2,397,730	Reference		Reference	
Pre-frail	7,954/2,745,987	1.79 (1.72, 1.86)	<0.001	1.59 (1.53, 1.65)	<0.001
Frail	1,703/311,522	3.44 (3.25, 3.64)	<0.001	3.14 (2.96, 3.34)	<0.001
Per 0.01-point increase		1.05 (1.05, 1.05)	<0.001	1.05 (1.05, 1.05)	<0.001
Per 0.05-point increase		1.29 (1.28, 1.30)	<0.001	1.28 (1.26, 1.29)	<0.001
Osteoarthritis					
Non-frail	20,993/2,282,881	Reference		Reference	
Pre-frail	36,593/2,550,775	1.57 (1.54, 1.59)	<0.001	1.32 (1.30, 1.34)	<0.001
Frail	6,904/275,572	2.76 (2.69, 2.84)	<0.001	1.90 (1.84, 1.95)	<0.001
Per 0.01-point increase		1.04 (1.04, 1.04)	<0.001	1.03 (1.03, 1.03)	<0.001
Per 0.05-point increase		1.23 (1.23, 1.24)	<0.001	1.14 (1.14, 1.15)	<0.001
Intervertebral disc degeneration					
Non-frail	3,129/2,398,197	Reference		Reference	
Pre-frail	6,216/2,748,677	1.74 (1.67, 1.81)	<0.001	1.61 (1.54, 1.68)	<0.001
Frail	1,397/311,604	3.47 (3.26, 3.70)	<0.001	2.82 (2.63, 3.01)	<0.001
Per 0.01-point increase		1.05 (1.05, 1.05)	<0.001	1.04 (1.04, 1.05)	<0.001
Per 0.05-point increase		1.29 (1.28, 1.30)	<0.001	1.24 (1.23, 1.26)	<0.001
Degenerative bone and joint multimorbidity					
Non-frail	2,076/2,409,189	Reference		Reference	
Pre-frail	5,289/2,764,308	2.25 (2.14, 2.37)	<0.001	1.85 (1.76, 1.95)	<0.001
Frail	1,444/313,902	5.59 (5.23, 5.98)	<0.001	3.89 (3.62, 4.18)	<0.001
Per 0.01-point increase		1.07 (1.07, 1.07)	<0.001	1.06 (1.06, 1.06)	<0.001
Per 0.05-point increase		1.41 (1.39, 1.43)	<0.001	1.33 (1.31, 1.34)	<0.001

PYs represents the person years from first or second follow-up to DBJDs onset. PYs, person years; TDI, Townsend deprivation index; BMI, body mass index. ^a^HR_1_ and P_1_ were unadjusted. ^b^HR_2_ and P_2_ were adjusted for age, sex, race, education, TDI, smoking status, drinking status, physical activity level and BMI.

When analyzed as a continuous variable, each 0.01-point increase in baseline FI was associated with higher risks of incident DBJDs (HR = 1.03, 95%CI = 1.03–1.03), OP (HR = 1.05, 95%CI = 1.05–1.05), OA (HR = 1.03, 95%CI = 1.03–1.03), IVDD (HR = 1.04, 95% CI: 1.04–1.05), and DBJM (HR = 1.06, 95%CI = 1.06–1.06). Consistent dose–response associations were observed when FI was analyzed per 0.05-point increase.

### 3.3 Frailty status transitions and incident DBJDs and their multimorbidity

The distribution of frailty status transitions between baseline and follow-up is presented in Table 2. Of the participants who were non-frail at baseline, 9,773 (35.3%) progressed to pre-frail or frail status, whereas 651 (42.8%) of those who were frail at baseline recovered to pre-frail or non-frail status. Over a median follow-up of 4.43 years, 4,045 incident DBJDs cases and 308 incident DBJM cases were identified.

**Table 2:** Number and percentage of the changes in frailty status.

Baseline survey	First or second follow-up	*N* (%)	DBJDs	DBJM
Non-frail	Non-frail	17,890 (33.7)	997	64
	Pre-frail	9,672 (18.2)	687	43
	Frail	101 (0.2)	5	0
Pre-frail	Non-frail	4,197 (7.9)	277	22
	Pre-frail	18,029 (33.9)	1,641	132
	Frail	1,774 (3.3)	216	21
Frail	Non-frail	12 (0.0)	2	0
	Pre-frail	639 (1.2)	83	10
	Frail	870 (1.6)	137	16

DBJDs, degenerative bone and joint diseases; DBJM, degenerative bone and joint multimorbidity.

**Figure 2:**
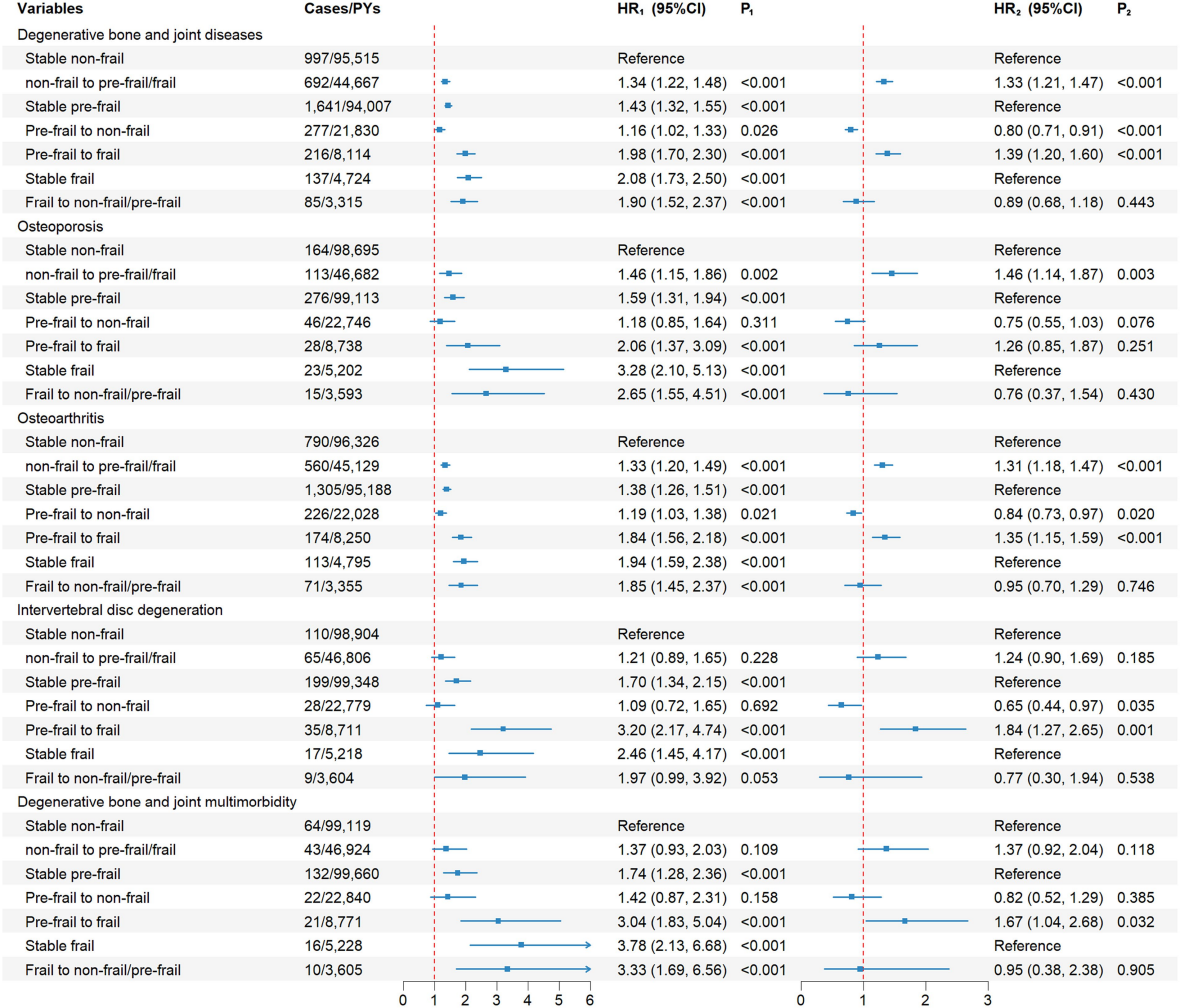
Association of changes in frailty status with the risk of incident degenerative bone and joint diseases and their multimorbidity. PYs, person years; HR, hazard ratio; CI, confidence interval; DBJDs, degenerative bone and joint diseases. Multivariable Cox proportional hazard models were used to estimate HRs and 95% CIs. Left panel (HR_1_, P_1_): The “Stable non-frail” group serves as the universal reference for all frailty transition categories. Right panel (HR_2_, P_2_): Analyses were stratified by baseline frailty status; transition groups were compared against their corresponding stable category (i.e., stable non-frail stable pre-frail, or stable frail) to evaluate the specific risk of frailty progression or recoverment. PYs represents the person years from the first or second follow-up to DBJDs onset.

Figure 2 shows the associations of changes in frailty status with the risks of incident DBJDs and their multimorbidity using two complementary reference frameworks. In the primary analysis, all transition groups were compared with participants who remained non-frail. Under this reference, remaining pre-frail (HR = 1.43, 95%CI = 1.32–1.55) or frail (HR = 2.08, 95%CI = 1.73–2.50) was associated with a markedly higher risk of DBJDs. The magnitude and significance of effects remained consistent for OP (HR = 1.59, 95%CI = 1.31–1.94; HR = 3.28, 95%CI = 2.10–5.13), OA (HR = 1.38, 95%CI = 1.26–1.51; HR = 1.94, 95%CI = 1.59–2.38) and IVDD (HR = 1.70, 95%CI = 1.34–2.15; HR = 2.46, 95%CI = 1.45–4.17), and became more pronounced for DBJM (HR = 1.74, 95%CI = 1.28–2.36; HR = 3.78, 95%CI = 2.13–6.68). Frailty progression was also associated with increased risk – participants progressed from non-frail to pre-frail or frail status showed elevated risks of DBJDs (HR = 1.34, 95%CI = 1.22–1.48), OP (HR = 1.46, 95%CI = 1.15–1.86) and OA (HR = 1.33, 95%CI = 1.20–1.49); participants progressed from pre-frail to frail status showed stronger and broader risk elevations, including DBJDs (HR = 1.98, 95%CI = 1.70–2.30), OP (HR = 2.06, 95%CI = 1.37–3.09), OA (HR = 1.84, 95%CI = 1.56–2.18), IVDD (HR = 3.20, 95%CI = 2.17–4.74) and DBJM (HR = 3.04, 95%CI = 1.83–5.04). In contrast, apparent recovery remained associated with higher risk compared with stable non-frail, such as pre-frail to non-frail for DBJDs (HR = 1.16, 95%CI = 1.02–1.33), and frail to non-frail/pre-frail for DBJDs (HR = 1.90, 95%CI = 1.52–2.37) and DBJM (HR = 3.33, 95%CI = 1.69–6.56).

In the secondary analysis, transition groups were compared with their corresponding stable categories according to baseline frailty status, providing a within-stratum estimate of progression or recovery. Under this framework, progression to a worse status remained associated with an increased risk: non-frail participants who progressed to pre-frail/frail status showed increased risks of DBJDs (HR = 1.33, 95%CI = 1.21–1.47), OP (HR = 1.46, 95%CI = 1.14–1.87), OA (HR = 1.31, 95%CI = 1.18–1.47) and pre-frail participants who progressed to frailty showed increased risks of DBJDs (HR = 1.39, 95%CI = 1.20–1.60), OA (HR = 1.35, 95%CI = 1.15–1.59), IVDD (HR = 1.84, 95%CI = 1.27–2.65) and DBJM (HR = 1.67, 95%CI = 1.04–2.68). In contrast, compared with stable pre-frail, recovery from pre-frailty to non-frailty was associated with lower risks of DBJDs (HR = 0.80, 95%CI = 0.71–0.91), OA (HR = 0.84, 95%CI = 0.73–0.97) and IVDD (HR = 0.65, 95%CI = 0.44–0.97). Although lacking statistical significance, recovery from frail to non-frail/pre-frail was associated with reduced risks of DBJDs (HR = 0.89, 95%CI = 0.68–1.18) and DBJM (HR = 0.95, 95%CI = 0.38–2.38) when compared with stable frailty.

### 3.4 Rate of change in frailty index and incident DBJDs and their multimorbidity

Table 3 shows the association of the rate of ΔFI with the risk of incident DBJDs and their multimorbidity. Participants with the highest tertile of the rate of ΔFI had significantly increased risk of incident DBJDs (HR = 1.34, 95%CI = 1.24–1.44; OP: HR = 1.56, 95%CI = 1.30–1.88; OA: HR = 1.27, 95%CI = 1.16–1.38; IVDD: HR = 1.34, 95%CI = 1.08–1.68) than those with the lowest tertile. Trend analyses indicated a monotonic increase in the risk of outcomes with increasing rates of ΔFI (all P_trend_ < 0.05). When analyzed as a continuous variable, each 0.01-point annual increase in ΔFI was associated with a significantly elevated risk of DBJDs (HR = 1.12, 95%CI = 1.08–1.15; OP: HR = 1.16, 95%CI = 1.07–1.25; OA: HR = 1.08, 95%CI = 1.05–1.12; IVDD: HR = 1.19, 95%CI = 1.09–1.31). Stronger associations were observed when ΔFI was analyzed per 0.05-point annual increase.

**Table 3:** Association of the rate of change in frailty index with the risk of incident degenerative bone and joint diseases and their multimorbidity.

Variables	Cases/PYs	HR_1_ (95%CI)^a^	P_1_^a^	HR_2_ (95%CI)^b^	P_2_^b^	HR_3_ (95%CI)^c^	P_3_^c^
Degenerative bone and joint diseases							
T1 of △FI/year	1,311/96,291	Reference		Reference		Reference	
T2 of △FI/year	1,086/79,062	1.02 (0.95, 1.11)	0.555	1.04 (0.96, 1.13)	0.325	1.18 (1.08, 1.28)	<0.001
T3 of △FI/year	1,648/96,818	1.25 (1.16, 1.34)	<0.001	1.17 (1.09, 1.26)	<0.001	1.34 (1.24, 1.44)	<0.001
*p* for trend test		<0.001		<0.001		<0.001	
△FI per 0.01-point/year increase		1.09 (1.05, 1.12)	<0.001	1.05 (1.02, 1.09)	0.002	1.12 (1.08, 1.15)	<0.001
△FI per 0.05-point/year increase		1.51 (1.28, 1.78)	<0.001	1.29 (1.10, 1.52)	0.002	1.73 (1.47, 2.03)	<0.001
Osteoporosis							
T1 of △FI/year	213/100,706	Reference		Reference		Reference	
T2 of △FI/year	173/82,089	1.05 (0.86, 1.28)	0.641	1.06 (0.87, 1.30)	0.551	1.28 (1.04, 1.57)	0.021
T3 of △FI/year	279/101,974	1.29 (1.08, 1.54)	0.006	1.28 (1.07, 1.53)	0.007	1.56 (1.30, 1.88)	<0.001
*p* for trend test		0.005		0.007		<0.001	
△FI per 0.01-point/year increase		1.06 (0.98, 1.15)	0.150	1.06 (0.98, 1.15)	0.126	1.16 (1.07, 1.25)	<0.001
△FI per 0.05-point/year increase		1.33 (0.90, 1.97)	0.150	1.36 (0.92, 2.00)	0.126	2.07 (1.41, 3.05)	<0.001
Osteoarthritis							
T1 of △FI/year	1,056/97,235	Reference		Reference		Reference	
T2 of △FI/year	884/79,693	1.04 (0.95, 1.13)	0.428	1.05 (0.96, 1.15)	0.244	1.17 (1.07, 1.29)	<0.001
T3 of △FI/year	1,299/98,144	1.22 (1.12, 1.32)	<0.001	1.13 (1.04, 1.22)	0.005	1.27 (1.16, 1.38)	<0.001
*p* for trend test		<0.001		0.005		<0.001	
△FI per 0.01-point/year increase		1.07 (1.03, 1.11)	<0.001	1.03 (0.99, 1.07)	0.106	1.08 (1.05, 1.12)	<0.001
△FI per 0.05-point/year increase		1.40 (1.17, 1.68)	<0.001	1.16 (0.97, 1.39)	0.106	1.50 (1.25, 1.80)	<0.001
Intervertebral disc degeneration							
T1 of △FI/year	161/100,820	Reference		Reference		Reference	
T2 of △FI/year	117/82,287	0.90 (0.71, 1.14)	0.389	0.91 (0.72, 1.16)	0.461	1.10 (0.86, 1.40)	0.450
T3 of △FI/year	185/102,265	1.13 (0.92, 1.40)	0.250	1.10 (0.89, 1.36)	0.372	1.34 (1.08, 1.68)	0.009
*p* for trend test		0.237		0.358		0.008	
△FI per 0.01-point/year increase		1.11 (1.01, 1.22)	0.031	1.09 (0.99, 1.20)	0.069	1.19 (1.09, 1.31)	<0.001
△FI per 0.05-point/year increase		1.70 (1.05, 2.75)	0.031	1.56 (0.97, 2.50)	0.069	2.42 (1.52, 3.85)	<0.001
Degenerative bone and joint multimorbidity							
T1 of △FI/year	116/101,087	Reference		Reference		Reference	
T2 of △FI/year	82/82,414	0.96 (0.72, 1.28)	0.786	0.98 (0.74, 1.31)	0.906	1.24 (0.93, 1.67)	0.142
T3 of △FI/year	110/102,644	0.92 (0.71, 1.20)	0.555	0.87 (0.67, 1.14)	0.312	1.14 (0.87, 1.49)	0.355
*p* for trend test		0.555		0.311		0.356	
△FI per 0.01-point/year increase		0.95 (0.85, 1.06)	0.319	0.93 (0.83, 1.03)	0.175	1.05 (0.94, 1.17)	0.369
△FI per 0.05-point/year increase		0.75 (0.43, 1.31)	0.319	0.69 (0.40, 1.18)	0.175	1.28 (0.75, 2.18)	0.369

△FI was calculated by the FI at the final assessment minus the FI at baseline. T1 was the lower tertile, T2 was the middle tertile, and T3 was the upper tertile. PYs represents the person years from first or second follow-up to DBJDs onset. FI, frailty index; PYs, person years; TDI, Townsend deprivation index; BMI, body mass index. ^a^HR_1_ and P_1_ were unadjusted. ^b^HR_2_ and P_2_ were adjusted for age, sex, race, education, TDI, smoking status, drinking status, physical activity level and BMI. ^c^HR_3_ and P_3_ were further adjusted for baseline FI based on Model 2.

### 3.5 Total frailty index and incident DBJDs and their multimorbidity

The associations of total FI with risk of incident DBJDs and DBJM are presented in Table 4. After multivariable adjustment, participants in the highest tertile of total FI had significantly increased risks of incident DBJDs (HR = 1.61, 95%CI = 1.49–1.75; OP: HR = 1.93, 95%CI = 1.58–2.36; OA: HR = 1.53, 95%CI = 1.39–1.67; IVDD: HR = 2.11, 95%CI = 1.65–2.70) and DBJM (HR = 2.24, 95%CI = 1.65–3.05) compared to those in the lowest tertile. When analyzed as a continuous variable, both 0.01-point and 0.05-point increases in total FI were associated with significantly elevated risks of DBJDs and DBJM.

**Table 4:** Association of total frailty index with the risk of incident degenerative bone and joint diseases and their multimorbidity.

Variables	Cases/PYs	HR_1_ (95%CI)^a^	P_1_^a^	HR_2_ (95%CI)^b^	P_2_^b^
Degenerative bone and joint diseases					
T1 of total FI	952/93,070	Reference		Reference	
T2 of total FI	1,293/89,334	1.42 (1.31, 1.54)	<0.001	1.28 (1.18, 1.39)	<0.001
T3 of total FI	1,800/89,767	1.96 (1.82, 2.12)	<0.001	1.61 (1.49, 1.75)	<0.001
*p* for trend test		<0.001		<0.001	
Per 0.01-point increase		1.02 (1.02, 1.02)	<0.001	1.02 (1.01, 1.02)	<0.001
Per 0.05-point increase		1.12 (1.10, 1.13)	<0.001	1.09 (1.07, 1.10)	<0.001
Osteoporosis					
T1 of total FI	151/96,106	Reference		Reference	
T2 of total FI	217/93,327	1.49 (1.21, 1.83)	<0.001	1.41 (1.14, 1.73)	0.001
T3 of total FI	297/95,336	1.98 (1.63, 2.41)	<0.001	1.93 (1.58, 2.36)	<0.001
*p* for trend test		<0.001		<0.001	
Per 0.01-point increase		1.02 (1.02, 1.03)	<0.001	1.02 (1.02, 1.03)	<0.001
Per 0.05-point increase		1.11 (1.08, 1.14)	<0.001	1.13 (1.10, 1.16)	<0.001
Osteoarthritis					
T1 of total FI	764/93,808	Reference		Reference	
T2 of total FI	1,032/90,300	1.41 (1.28, 1.54)	<0.001	1.25 (1.13, 1.37)	<0.001
T3 of total FI	1,443/90,964	1.95 (1.79, 2.13)	<0.001	1.53 (1.39, 1.67)	<0.001
*p* for trend test		<0.001		<0.001	
Per 0.01-point increase		1.02 (1.02, 1.02)	<0.001	1.01 (1.01, 1.02)	<0.001
Per 0.05-point increase		1.12 (1.10, 1.13)	<0.001	1.07 (1.06, 1.09)	<0.001
Intervertebral disc degeneration					
T1 of total FI	99/96,312	Reference		Reference	
T2 of total FI	138/93,516	1.44 (1.11, 1.86)	0.006	1.39 (1.07, 1.80)	0.013
T3 of total FI	226/95,543	2.30 (1.82, 2.92)	<0.001	2.11 (1.65, 2.70)	<0.001
*p* for trend test		<0.001		<0.001	
Per 0.01-point increase		1.03 (1.02, 1.03)	<0.001	1.03 (1.02, 1.03)	<0.001
Per 0.05-point increase		1.15 (1.12, 1.19)	<0.001	1.14 (1.10, 1.17)	<0.001
Degenerative bone and joint multimorbidity					
T1 of total FI	59/96,495	Reference		Reference	
T2 of total FI	88/93,810	1.55 (1.12, 2.16)	0.009	1.38 (0.99, 1.93)	0.057
T3 of total FI	161/95,840	2.74 (2.03, 3.69)	<0.001	2.24 (1.65, 3.05)	<0.001
*p* for trend test		<0.001		<0.001	
Per 0.01-point increase		1.03 (1.03, 1.04)	<0.001	1.03 (1.02, 1.04)	<0.001
Per 0.05-point increase		1.19 (1.14, 1.23)	<0.001	1.17 (1.12, 1.22)	<0.001

Total FI was calculated by the FI at baseline plus the FI at the final assessment. T1 was the lower tertile, T2 was the middle tertile, and T3 was the upper tertile. PYs represents the person years from first or second follow-up to DBJDs onset. FI, frailty index; PYs, person years; TDI, Townsend deprivation index; BMI, body mass index; DBJDs, degenerative bone and joint diseases. ^a^HR_1_ and P_1_ were unadjusted. ^b^HR2 and P2 were adjusted for age, sex, race, education, TDI, smoking status, drinking status, physical activity level and BMI.

### 3.6 Stratified and sensitivity analyses

The associations between frailty and the risks of DBJDs and DBJM remained consistent with the primary analysis in stratified analyses by sex (males and females, Supplementary Tables S4–S7) and age group (<60 and ≥60 years, Supplementary Tables S8–S11). Sensitivity analyses further supported the robustness of findings. Similar results were observed when using another two FI cut-off values (Supplementary Tables S12, S13). Meanwhile, the results were not changed when excluding cases occurring within the first one or 2 years of follow-up (Supplementary Tables S14–S19).

## 4 Discussion

In this prospective cohort study, we found that baseline frailty status and its longitudinal changes were strongly associated with the risk of incident DBJDs, including OP, OA, IVDD, as well as their multimorbidity. Beyond baseline frailty, dynamic frailty measures—captured by frailty status transitions, the rate of change in frailty index, and cumulative frailty burden—provided additional and consistent prognostic information. Frailty progression and cumulative burden were associated with substantially higher risks of DBJDs and DBJM, whereas recovery from pre-frailty to non-frailty mitigated these risks. Together, these findings highlight frailty as a dynamic integrative marker of musculoskeletal vulnerability and disease accumulation across midlife and older age.

Previous studies have reported associations between frailty and musculoskeletal health (40–42), providing an important foundation for understanding the link between systemic vulnerability and skeletal disorders. For example, Cook et al., conducted a longitudinal analysis of 3,231 men aged 40–79 years, revealing that the skeletal sites associated with frailty differed depending on how frailty was operationalized: frailty assessed using the Fried phenotype was associated with lower femoral neck bone mineral density (BMD), whereas frailty assessed using a frailty index was associated with lower lumbar spine BMD (21). While these studies consistently indicate that frailty is linked to adverse skeletal outcomes, they have largely focused on baseline status, structural bone measures, or single musculoskeletal endpoints. In contrast, our study extends existing literature in several important ways. First, we examined clinically diagnosed DBJDs rather than merely surrogate markers such as BMD, thereby capturing outcomes that are of high clinical relevance. Second, we evaluated DBJM, addressing disease accumulation that more accurately reflects real-world aging trajectories. Third and the most importantly, we incorporated longitudinal frailty dynamics, showing that changes over time convey important prognostic information beyond simply baseline level.

A key finding of our study is that frailty progression was consistently associated with increased risks of DBJDs and their multimorbidity across multiple analytical frameworks. Only limited evidence has previously addressed this question. In a cohort of 1,044 community-dwelling older women, Bartosch et al. observed a 6–7% annual increase in frailty over 10 years of follow-up, which was associated with accelerated femoral neck BMD decline (26). Expanding this evidence, our study showed that frailty is a dynamic process characterized by transitions between frailty states, and that worsening frailty over time was associated with elevated risks of adverse musculoskeletal outcomes. Crucially, participants who progressed from non-frail to pre-frail or frail status already exhibited meaningful increases in risk, whereas those who progressed from pre-frail to frail status experienced the greatest risk elevations, particularly for IVDD and multimorbidity. This graded risk pattern supports a dose–response relationship between worsening systemic vulnerability and degenerative musculoskeletal outcomes. The pronounced susceptibility of the spine to frailty progression likely reflects the unique biomechanical and metabolic demands of spinal structures. Consistent with these transition-based findings, both the rate of frailty progression and cumulative frailty burden were independently associated with incident DBJDs and DBJM. A faster increase in frailty index over time was monotonically associated with higher disease risk, even after accounting for baseline frailty level, while greater total frailty burden conferred substantially elevated risks across all outcomes. Together, these findings reinforce the concept that frailty captures cumulative multisystem dysregulation and biological aging processes that contribute to musculoskeletal degeneration over the life course.

Notably, frailty recovery was associated with a lower risk of DBJDs and their multimorbidity, although statistical significance was primarily observed for recovery from pre-frailty to non-frailty. A similar but non-significant trend toward risk reduction was also evident among individuals recovering from frailty to pre-frailty or non-frailty. These findings suggest that pre-frailty may represent a critical window for intervention, during which reversing early frailty trajectories could potentially reduce the risk of incident musculoskeletal conditions. Although frailty status may improve over time, underlying biological or structural damage within the musculoskeletal system may be not fully reversible, particularly after progression to frailty status. Alternatively, improvements captured by the FI may reflect partial recovery of functional deficits that is insufficient to offset the long-term cumulative processes driving musculoskeletal degeneration.

Several biological and functional pathways may underline the observed associations, including chronic inflammation, cellular senescence, mitochondrial dysfunction and deregulated nutrient sensing (5, 43–45). These processes are central to both frailty and musculoskeletal aging and may contribute to impaired tissue repair, structural degeneration, and functional decline. Although mechanistic pathways were not directly examined in this study, the consistency of associations across multiple frailty metrics and disease outcomes supports shared multisystem mechanism linking frailty progression to musculoskeletal degeneration. The pronounced susceptibility of the spine to frailty progression may reflect the unique biomechanical loading and metabolic demands of spinal structures.

Our findings have important clinical and public health implications. Frailty has been increasingly recognized as a potentially modifiable condition, with assessment feasible in both clinical and population-based settings. Our results suggest that monitoring frailty trajectories, rather than relying solely on a single baseline assessment, may help identify individuals at elevated risk of developing DBJDs and multimorbidity. Importantly, our study highlights the value of targeting pre-frailty as a critical window for the early identification and prevention of frailty progression, particularly during midlife and early older age, when interventions may exert greater long-term benefits.

This study has several strengths, including its large sample size, prospective design, long follow-up duration, and comprehensive assessment of frailty dynamics using both categorical and continuous measures. The simultaneous evaluation of single DBJDs and their multimorbidity provides clinically relevant insights into disease accumulation in aging populations. Nevertheless, several limitations should be considered. First, findings on frailty recovery should be interpreted cautiously due to the small proportion of improving participants. Consequently, the observed null associations may reflect limited statistical power. Larger cohorts are needed to validate the protective benefits of frailty recovery. Second, frailty components were derived largely from self-report, which might lead to a misclassification bias, although the FI used has been extensively validated. Third, UKB participants are generally healthier than the general population, which may limit generalizability of absolute risk estimates, although relative associations are likely robust. Fourth, although we have adjusted for multiple confounders, residual confounders might remain, such as genetic susceptibility and diet. Finally, because of the intrinsic limitations of observational research, no direct causation can be drawn. The underlying causality and mechanisms are worthy of further research.

## 5 Conclusion

Frailty status and, importantly, its longitudinal changes are strongly associated with the risk of incident DBJDs and DBJM. Frailty progression and cumulative burden confer substantially increased musculoskeletal risks, whereas evidence for risk reduction associated with frailty recovery remains limited and less consistent. These findings underscore the importance of viewing frailty as a dynamic marker of musculoskeletal vulnerability and suggest that pre-frailty may represent a critical window for early intervention. Preventing frailty progression at this stage may help reduce the burden of DBJDs and their multimorbidity in aging populations.

## Data Availability

Publicly available datasets were analyzed in this study. This data can be found at: https://www.ukbiobank.ac.uk/.
