# Supplementary material for: Changes in frailty and incident risk of degenerative bone and joint diseases and their multimorbidity: a prospective cohort study
Source: Front Public Health. 2026 May 8;14:1831566. doi: 10.3389/fpubh.2026.1831566 (PMC13194387; doi:10.3389/fpubh.2026.1831566)
Supplement: Supplementary file 1 [file Data_Sheet_1.pdf]

Table S1. The 36 items used to construct the frailty index.

Type of deficit	No	Description of the item	Cut-off value
Sensory	1	Glaucoma	Yes = 1<br>No = 0
Sensory	2	Cataracts	
Sensory	3	Hearing difficulty/problems	Yes = 1<br>I am completely deaf = 1<br>No = 0
Cranial	4	Migraine	Yes = 1<br>No = 0
Cranial	5	Mouth/teeth dental problems	Any = 1<br>None of the above = 0
Mental wellbeing	6	Overall health rating	Poor = 1<br>Fair = 0.5<br>Good = 0.25<br>Excellent = 0
Mental wellbeing	7	Frequency of tiredness/lethargy in last two weeks	Nearly every day = 1<br>More than half the days = 0.5<br>Several days = 0.25<br>None = 0
Mental wellbeing	8	Sleeplessness/insomnia	Usually = 1<br>Sometimes = 0.5<br>Never/rarely = 0
Mental wellbeing	9	Frequency of depressed mood in last two weeks	Nearly every day = 1<br>More than half the days = 0.75<br>Several days = 0.5<br>Not at all = 0
Mental wellbeing	10	Nervous feelings	Yes = 1<br>No = 0
Mental wellbeing	11	Anxiety / panic attacks	
Mental wellbeing	12	Loneliness, isolation	
Mental wellbeing	13	Miserableness	
Infirmity	14	Long-standing illness, disability or infirmity	Yes = 1<br>No = 0

Infirmity	15	Falls in the last year	More than one fall = 1<br>Only one fall = 0.5<br>No falls = 0
Infirmity	16	Fractured/broken bones in last five years	Yes = 1<br>No = 0
Cardiometabolic	17	Diabetes	Yes = 1<br>No = 0
Cardiometabolic	18	Myocardial infarction	
Cardiometabolic	19	Angina	
Cardiometabolic	20	Stroke /ischaemic stroke	
Cardiometabolic	21	Hypertension	
Cardiometabolic	22	Hypothyroidism	
Cardiometabolic	23	Deep venous thrombosis	
Cardiometabolic	24	Cholesterol lowering medication use	
Respiratory	25	Wheeze or whistling in the chest in last year	Yes = 1<br>No = 0
Respiratory	26	Pneumonia	
Respiratory	27	Emphysema / chronic bronchitis	
Respiratory	28	Asthma	
Immunological	29	Hay fever / allergic rhinitis or eczema/dermatitis	Yes = 1<br>No = 0
Immunological	30	Psoriasis	
Cancer	31	Any cancer diagnosis	At least 1 = 1<br>Yes = 1<br>No = 0
Cancer	32	Multiple cancers diagnosed	More than 1 = 1<br>0 or 1 = 0
Gastrointestinal	33	Gastro-oesophageal reflux / gastric reflux	Yes = 1<br>No = 0
Gastrointestinal	34	Hiatus hernia	
Gastrointestinal	35	Cholelithiasis / gall stones	
Gastrointestinal	36	Diverticular disease / diverticulitis	

Table S2. Baseline characteristics of participants for baseline frailty status analyses.

Variables	Baseline survey		
	Non-frail	Pre-frail	Frail
Number, n (%)	179,440 (43.4)	210,571 (50.7)	25,470 (5.9)
Age, mean (SD), years	55.82 (8.14)	54.72 (8.10)	57.84 (7.74)
Sex, n (%)			
Female	91,631 (51.1)	115,148 (54.7)	13,426 (52.7)
Male	87,809 (48.9)	95,423 (45.3)	12,044 (47.3)
Ethnicity, n (%)			
White	169,673 (94.6)	198,490 (94.3)	23,658 (92.9)
Other	9,767 (5.4)	12,081 (5.7)	1,812 (7.1)
Education, n (%)			
Less than college or university degree	77,998 (43.5)	98,128 (46.6)	12,927 (50.8)
High education level	101,442 (56.5)	112,443 (53.4)	12,543 (49.2)
Townsend deprivation index, n (%)			
T1	65,659 (36.6)	67,415 (32.0)	5,378 (21.1)
T2	62,896 (35.1)	71,341 (33.9)	7,281 (28.6)
T3	50,885 (28.3)	71,815 (34.1)	12,811 (50.3)
Smoking status, n (%)			
Never	109,333 (60.9)	111,028 (52.7)	10,504 (41.2)
Previous	54,589 (30.4)	75,614 (35.9)	10,151 (39.9)
Current	15,518 (8.7)	23,929 (11.4)	4,815 (18.9)
Alcohol drinking status, n (%)			
Never	6,848 (3.8)	9,139 (4.3)	1,707 (6.7)
Previous	3,855 (2.2)	7,830 (3.7)	2,197 (8.6)
Current	168,737 (94.0)	193,602 (92.0)	21,566 (84.7)
Physical activity level, n (%)			
Low	27,838 (15.5)	41,326 (19.6)	7,531 (29.6)
Moderate	72,616 (40.5)	87,176 (41.4)	9,972 (39.1)
High	78,986 (44.0)	82,069 (39.0)	7,967 (31.3)
BMI, mean (SD), kg/m^2	26.39 (4.07)	27.57 (4.78)	30.00 (5.94)

Continuous variables are presented as mean (SD), and categorical variables are presented as number (percentage).

Abbreviations: SD, standard deviation; BMI, body mass index.

Table S3. Baseline characteristics of participants for changes in frailty status analyses

	Baseline survey		
**Variables**	**Non-frail**	**Pre-frail**	**Frail**
Number, n (%)	27,663 (52.0)	24,000 (45.1)	1,521 (2.9)
Age, mean (SD), years	54.17 (7.63)	55.22 (7.69)	56.24 (7.50)
Sex, n (%)			
Female	13,261 (47.9)	12,349 (51.5)	760 (50.0)
Male	14,402 (52.1)	11,651 (48.5)	761 (50.0)
Ethnicity, n (%)			
White	26,789 (96.8)	23,248 (96.9)	1,465 (96.3)
Other	874 (3.2)	752 (3.1)	56 (3.7)
Education, n (%)			
Less than college or university degree	10,362 (37.5)	9,880 (41.2)	680 (44.7)
High education level	17,301 (62.5)	14,120 (58.8)	841 (55.3)
Townsend deprivation index, n (%)			
T1	11,313 (40.9)	9,092 (37.9)	457 (30.0)
T2	9,742 (35.2)	8,402 (35.0)	517 (34.0)
T3	6,608 (23.9)	6,506 (27.1)	547 (36.0)
Smoking status, n (%)			
Never	17,792 (64.3)	13,840 (57.7)	777 (51.1)
Previous	8,267 (29.9)	8,393 (35.0)	600 (39.4)
Current	3,515 (5.8)	1,767 (7.3)	144 (9.5)
Alcohol drinking status, n (%)			
Never	713 (2.6)	619 (2.6)	50 (3.3)
Previous	428 (1.5)	677 (2.8)	86 (5.7)
Current	26,522 (95.9)	22,704 (94.6)	1,385 (91.0)
Physical activity level, n (%)			
Low	4,490 (16.2)	4,826 (20.1)	438 (28.8)
Moderate	11,461 (41.4)	10,288 (42.9)	612 (40.2)
High	11,712 (42.4)	8,886 (37.0)	471 (31.0)
BMI, mean (SD), kg/m^2	25.97 (3.81)	26.99 (4.36)	29.46 (5.34)

Continuous variables are presented as mean (SD), and categorical variables are presented as number (percentage).

Abbreviations: SD, standard deviation; BMI, body mass index.

Table S4. Association of changes in frailty status with the risk of incident degenerative bone and joint diseases and its multimorbidity stratified by sex, using the "Stable non-frail group" as reference.

Variables	Cases/PYs	HR(95%CI)[a]	P[a]
**Degenerative bone and joint diseases**			
Male			
Stable non-frail	458/49,836	Reference	
Non-frail to pre-frail/frail	339/23,758	1.35 (1.17, 1.55)	<0.001
Stable pre-frail	709/46,807	1.38 (1.22, 1.56)	<0.001
Pre-frail to non-frail	105/10,215	1.09 (0.88, 1.35)	0.419
Pre-frail to frail	73/4,097	1.49 (1.16, 1.91)	0.002
Stable frail	62/2,370	2.11 (1.61, 2.76)	<0.001
Frail to non-frail/pre-frail	40/1,672	1.97 (1.42, 2.73)	<0.001
Female			
Stable non-frail	539/45,678	Reference	
Non-frail to pre-frail/frail	353/20,909	1.33 (1.16, 1.52)	<0.001
Stable pre-frail	932/47,200	1.48 (1.33, 1.65)	<0.001
Pre-frail to non-frail	172/11,615	1.21 (1.02, 1.44)	0.027
Pre-frail to frail	143/4,017	2.39 (1.98, 2.89)	<0.001
Stable frail	75/2,354	2.07 (1.61, 2.66)	<0.001
Frail to non-frail/pre-frail	45/1,643	1.84 (1.36, 2.50)	<0.001
P for interaction		0.135	
**Osteoporosis**			
Male			
Stable non-frail	27/51,431	Reference	
Non-frail to pre-frail/frail	29/24,774	2.05 (1.20, 3.50)	0.009
Stable pre-frail	58/49,211	1.97 (1.23, 3.16)	0.005
Pre-frail to non-frail	4/10,574	0.76 (0.26, 2.19)	0.606
Pre-frail to frail	4/4,315	1.56 (0.54, 4.56)	0.410
Stable frail	8/2,614	5.20 (1.30, 11.13)	<0.001
Frail to non-frail/pre-frail	4/1,792	3.80 (2.28, 11.82)	0.015
Female			
Stable non-frail	137/47,264	Reference	
Non-frail to pre-frail/frail	84/21,908	1.33 (1.01, 1.75)	0.042
Stable pre-frail	218/49,903	1.51 (1.21, 1.87)	<0.001
Pre-frail to non-frail	42/12,172	1.24 (0.88, 1.76)	0.220
Pre-frail to frail	24/4,423	2.16 (1.39, 3.36)	0.030
Stable frail	15/2,588	2.75 (1.59, 4.74)	<0.001
Frail to non-frail/pre-frail	11/1,801	2.39 (1.29, 4.44)	0.006
P for interaction		0.305	
**Osteoarthritis**			
Male			
Stable non-frail	389/50,089	Reference	
Non-frail to pre-frail/frail	292/23,915	1.34 (1.15, 1.56)	<0.001

Stable pre-frail	595/47,159	1.32 (1.16, 1.51)	<0.001
Pre-frail to non-frail	92/10,268	1.12 (0.89, 1.40)	0.337
Pre-frail to frail	61/4,135	1.40 (1.07, 1.84)	0.016
Stable frail	52/2,410	1.97 (1.46, 2.64)	<0.001
Frail to non-frail/pre-frail	36/1,681	2.00 (1.42, 2.83)	<0.001
Female			
Stable non-frail	401/46,237	Reference	
Non-frail to pre-frail/frail	268/21,214	1.32 (1.13, 1.54)	<0.001
Stable pre-frail	710/48,029	1.44 (1.27, 1.63)	0.024
Pre-frail to non-frail	134/11,760	1.25 (1.03, 1.52)	<0.001
Pre-frail to frail	113/4,115	2.24 (1.80, 2.77)	<0.001
Stable frail	61/2,385	1.92 (1.45, 2.54)	<0.001
Frail to non-frail/pre-frail	35/1,674	1.73 (1.22, 2.45)	0.002
P for interaction		0.165	
**Intervertebral disc degeneration**			
Male			
Stable non-frail	63/51,280	Reference	
Non-frail to pre-frail/frail	32/24,762	1.00 (0.65, 1.54)	0.998
Stable pre-frail	97/49,019	1.52 (1.09, 2.10)	0.013
Pre-frail to non-frail	14/10,534	1.06 (0.59, 1.90)	0.844
Pre-frail to frail	15/4,292	2.51 (1.41, 4.48)	0.002
Stable frail	10/2,601	2.71 (1.36, 5.40)	0.005
Frail to non-frail/pre-frail	4/1,786	1.63 (0.58, 4.54)	0.349
Female			
Stable non-frail	47/47,624	Reference	
Non-frail to pre-frail/frail	33/22,044	1.50 (0.96, 2.35)	0.078
Stable pre-frail	102/50,329	1.94 (1.36, 2.76)	<0.001
Pre-frail to non-frail	14/12,245	1.13 (0.62, 2.06)	0.686
Pre-frail to frail	20/4,419	4.05 (2.36, 6.96)	<0.001
Stable frail	7/2,617	2.21 (0.97, 5.04)	0.058
Frail to non-frail/pre-frail	5/1,818	2.43 (0.95, 6.21)	0.063
P for interaction		0.790	
**Degenerative bone and joint multimorbidity**			
Male			
Stable non-frail	21/51,455	Reference	
Non-frail to pre-frail/frail	14/24,830	1.22 (0.61, 2.45)	0.564
Stable pre-frail	38/49,240	1.54 (0.88, 2.68)	0.125
Pre-frail to non-frail	5/10,577	1.14 (0.42, 3.07)	0.797
Pre-frail to frail	7/4,318	3.20 (1.32, 7.77)	0.011
Stable frail	8/2,618	5.65 (2.39, 13.34)	<0.001
Frail to non-frail/pre-frail	4/1,790	4.30 (1.42, 13.02)	0.010
Female			
Stable non-frail	43/47,664	Reference	
Non-frail to pre-frail/frail	29/22,093	1.47 (0.91, 2.36)	0.115

Stable pre-frail	94/50,420	1.83 (1.27, 2.65)	0.001
Pre-frail to non-frail	17/12,263	1.53 (0.87, 2.69)	0.142
Pre-frail to frail	14/4,452	2.99 (1.60, 5.56)	<0.001
Stable frail	8/2,610	2.83 (1.29, 6.22)	0.010
Frail to non-frail/pre-frail	6/1,815	2.95 (1.24, 7.04)	0.015
P for interaction		0.763	

PYs represents the person years from first or second follow-up to DBJDs onset.

Abbreviations: PYs, person years; TDI, Townsend deprivation index; BMI, body mass index.

[a]HR and P were adjusted for age, sex, race, education, TDI, smoking status, drinking status, physical activity level and BMI.

Table S5. Association of changes in frailty status with the risk of incident degenerative bone and joint diseases and its multimorbidity stratified by sex, using each stable frailty status group as its own reference.

Variables	Cases/PYs	HR(95%CI)[a]	P[a]
**Degenerative bone and joint diseases**			
Male			
Stable non-frail	458/49,836	Reference	
Non-frail to pre-frail/frail	339/23,758	1.35 (1.17, 1.56)	<0.001
Female			
Stable non-frail	539/45,678	Reference	
Non-frail to pre-frail/frail	353/20,909	1.31 (1.14, 1.50)	<0.001
P for interaction		0.877	
Male			
Stable pre-frail	709/46,807	Reference	
Pre-frail to non-frail	105/10,215	0.78 (0.64, 0.96)	0.022
Pre-frail to frail	73/4,097	1.07 (0.84, 1.37)	0.567
Female			
Stable pre-frail	932/47,200	Reference	
Pre-frail to non-frail	172/11,615	0.81 (0.69, 0.96)	0.014
Pre-frail to frail	143/4,017	1.63 (1.36, 1.95)	<0.001
P for interaction		0.043	
Male			
Stable frail	62/2,370	Reference	
Frail to non-frail/pre-frail	40/1,672	0.93 (0.62, 1.40)	0.719
Female			
Stable frail	75/2,354	Reference	
Frail to non-frail/pre-frail	45/1,643	0.89 (0.60, 1.31)	0.544
P for interaction		0.981	
**Osteoporosis**			
Male			
Stable non-frail	27/51,431	Reference	
Non-frail to pre-frail/frail	29/24,774	2.14 (1.23, 3.74)	0.008
Female			
Stable non-frail	137/47,264	Reference	
Non-frail to pre-frail/frail	84/21,908	1.31 (0.99, 1.73)	0.056
P for interaction		0.205	
Male			
Stable pre-frail	58/49,211	Reference	
Pre-frail to non-frail	4/10,574	0.38 (0.13, 1.08)	0.070
Pre-frail to frail	4/4,315	0.74 (0.26, 2.11)	0.573
Female			
Stable pre-frail	218/49,903	Reference	
Pre-frail to non-frail	42/12,172	0.84 (0.60, 1.17)	0.292
Pre-frail to frail	24/4,423	1.40 (0.91, 2.15)	0.122

P for interaction		0.226	
Male			
Stable frail	5/3,148	Reference	
Frail to non-frail/pre-frail	5/2,825	0.76 (0.22, 2.62)	0.664
Female			
Stable frail	15/2,588	Reference	
Frail to non-frail/pre-frail	11/1,801	0.76 (0.30, 1.89)	0.515
P for interaction		0.787	
**Osteoarthritis**			
Male			
Stable non-frail	389/50,089	Reference	
Non-frail to pre-frail/frail	292/23,915	1.32 (1.13, 1.55)	<0.001
Female			
Stable non-frail	401/46,237	Reference	
Non-frail to pre-frail/frail	268/21,214	1.30 (1.11, 1.52)	0.001
P for interaction		0.990	
Male			
Stable pre-frail	595/47,159	Reference	
Pre-frail to non-frail	92/10,268	0.83 (0.66, 1.03)	0.097
Pre-frail to frail	61/4,135	1.06 (0.81, 1.38)	0.662
Female			
Stable pre-frail	710/48,029	Reference	
Pre-frail to non-frail	134/11,760	0.86 (0.71, 1.03)	0.103
Pre-frail to frail	113/4,115	1.59 (1.30, 1.94)	<0.001
P for interaction		0.070	
Male			
Stable frail	52/2,410	Reference	
Frail to non-frail/pre-frail	36/1,681	1.01 (0.65, 1.57)	0.955
Female			
Stable frail	61/2,385	Reference	
Frail to non-frail/pre-frail	35/1,674	0.94 (0.61, 1.46)	0.793
P for interaction		0.799	
**Intervertebral disc degeneration**			
Male			
Stable non-frail	63/51,280	Reference	
Non-frail to pre-frail/frail	32/24,762	1.07 (0.69, 1.67)	0.691
Female			
Stable non-frail	47/47,624	Reference	
Non-frail to pre-frail/frail	33/22,044	1.49 (0.94, 2.35)	0.090
P for interaction		0.306	
Male			
Stable pre-frail	97/49,019	Reference	
Pre-frail to non-frail	14/10,534	0.73 (0.41, 1.30)	0.282
Pre-frail to frail	15/4,292	1.58 (0.91, 2.76)	0.104

Female			
Stable pre-frail	102/50,329	Reference	
Pre-frail to non-frail	14/12,245	0.58 (0.33, 1.03)	0.061
Pre-frail to frail	20/4,419	2.07 (1.26, 3.38)	0.004
P for interaction		0.725	
Male			
Stable frail	10/2,601	Reference	
Frail to non-frail/pre-frail	4/1,786	0.69 (0.22, 2.18)	0.526
Female			
Stable frail	7/2,617	Reference	
Frail to non-frail/pre-frail	5/1,818	0.92 (0.28, 3.05)	0.895
P for interaction		0.684	
**Degenerative bone and joint multimorbidity**			
Male			
Stable non-frail	21/51,455	Reference	
Non-frail to pre-frail/frail	14/24,830	1.26 (0.60, 2.64)	0.516
Female			
Stable non-frail	43/47,664	Reference	
Non-frail to pre-frail/frail	29/22,093	1.45 (0.89, 2.36)	0.131
P for interaction		0.745	
Male			
Stable pre-frail	38/49,240	Reference	
Pre-frail to non-frail	5/10,577	0.74 (0.28, 1.97)	0.540
Pre-frail to frail	7/4,318	1.97 (0.85, 4.58)	0.848
Female			
Stable pre-frail	94/50,420	Reference	
Pre-frail to non-frail	17/12,263	0.83 (0.49, 1.41)	0.493
Pre-frail to frail	14/4,452	1.57 (0.88, 2.80)	0.124
P for interaction		0.802	
Male			
Stable frail	8/2,618	Reference	
Frail to non-frail/pre-frail	4/1,790	0.90 (0.27, 3.01)	0.864
Female			
Stable frail	8/2,610	Reference	
Frail to non-frail/pre-frail	6/1,815	1.21 (0.39, 3.75)	0.746
P for interaction		0.843	

PYs represents the person years from first or second follow-up to DBJDs onset.

Abbreviations: PYs, person years; TDI, Townsend deprivation index; BMI, body mass index.

[a]HR and P were adjusted for age, sex, race, education, TDI, smoking status, drinking status, physical activity level and BMI.

Table S6. Association of the rate of change in frailty index with incident degenerative bone and joint diseases and its multimorbidity stratified by sex.

Variables	Cases/PYs	HR(95%CI)[a]	P[a]
**Degenerative bone and joint diseases**			
Male			
T1 of △FI / year	539/46,846	Reference	
T2 of △FI / year	484/40,842	1.16 (1.02, 1.32)	0.022
T3 of △FI / year	885/45,752	1.32 (1.17, 1.48)	<0.001
P for trend test		<0.001	
Female			
T1 of △FI / year	772/49,445	Reference	
T2 of △FI / year	602/38,220	1.19 (1.07, 1.33)	0.002
T3 of △FI / year	578/29,724	1.36 (1.23, 1.50)	<0.001
P for trend test		<0.001	
P for interaction		0.951	
**Osteoporosis**			
Male			
T1 of △FI / year	32/48,853	Reference	
T2 of △FI / year	35/42,270	1.68 (1.02, 2.76)	0.042
T3 of △FI / year	67/53,588	2.15 (1.38, 3.36)	<0.001
P for trend test		<0.001	
Female			
T1 of △FI / year	181/51,854	Reference	
T2 of △FI / year	138/39,818	1.20 (0.96, 1.51)	0.116
T3 of △FI / year	212/48,386	1.45 (1.18, 1.78)	<0.001
P for trend test		<0.001	
P for interaction		0.339	
**Osteoarthritis**			
Male			
T1 of △FI / year	461/47,136	Reference	
T2 of △FI / year	418/41,038	1.16 (1.01, 1.33)	0.034
T3 of △FI / year	638/51,484	1.26 (1.11, 1.42)	<0.001
P for trend test		<0.001	
Female			
T1 of △FI / year	595/50,100	Reference	
T2 of △FI / year	466/38,655	1.19 (1.05, 1.35)	0.007
T3 of △FI / year	661/46,660	1.27 (1.13, 1.42)	<0.001
P for trend test		<0.001	
P for interaction		0.869	
**Intervertebral disc degeneration**			
Male			
T1 of △FI / year	78/48,652	Reference	
T2 of △FI / year	60/42,142	1.07 (0.76, 1.52)	0.690
T3 of △FI / year	97/53,482	1.33 (0.97, 1.82)	0.072

P for trend test		0.067	
Female			
T1 of △FI / year	83/52,168	Reference	
T2 of △FI / year	57/40,145	1.13 (0.80, 1.61)	0.478
T3 of △FI / year	88/48,783	1.36 (1.00, 1.87)	0.052
P for trend test		0.051	
P for interaction		0.997	
**Degenerative bone and joint multimorbidity**			
Male			
T1 of △FI / year	32/48,859	Reference	
T2 of △FI / year	27/42,279	1.37 (0.80, 2.34)	0.243
T3 of △FI / year	38/53,691	1.30 (0.79, 2.14)	0.291
P for trend test		0.305	
Female			
T1 of △FI / year	84/52,228	Reference	
T2 of △FI / year	55/40,135	1.20 (0.84, 1.70)	0.316
T3 of △FI / year	72/48,954	1.08 (0.78, 1.50)	0.647
P for trend test		0.637	
P for interaction		0.907	

△FI was calculated by the FI at the final assessment minus the FI at baseline. T1 was the lower tertile, T2 was the middle tertile, and T3 was the upper tertile. PYs represents the person years from first or second follow-up to DBJDs onset.

Abbreviations: PYs, person years; TDI, Townsend deprivation index; BMI, body mass index. aHR and P were adjusted for age, sex, race, education, TDI, smoking status, drinking status, physical activity level, BMI and baseline FI.

Table S7. Association of total frailty index with incident degenerative bone and joint diseases and its multimorbidity stratified by sex.

Variables	Cases/PYs	HR(95%CI)[a]	P[a]
**Degenerative bone and joint diseases**			
Male			
T1 of total FI	446/48,737	Reference	
T2 of total FI	569/45,098	1.24 (1.09, 1.40)	<0.001
T3 of total FI	771/44,919	1.51 (1.33, 1.70)	<0.001
P for trend test		<0.001	
Female			
T1 of total FI	506/44,333	Reference	
T2 of total FI	724/44,235	1.32 (1.18, 1.48)	<0.001
T3 of total FI	1,029/44,848	1.71 (1.53, 1.91)	<0.001
P for trend test		<0.001	
P for interaction		0.410	
**Osteoporosis**			
Male			
T1 of total FI	26/50,262	Reference	
T2 of total FI	45/46,910	1.73 (1.06, 2.83)	0.029
T3 of total FI	63/47,538	2.18 (1.36, 3.51)	0.001
P for trend test		0.002	
Female			
T1 of total FI	125/45,843	Reference	
T2 of total FI	172/46,417	1.34 (1.06, 1.68)	0.015
T3 of total FI	234/47,798	1.86 (1.49, 2.33)	<0.001
P for trend test		<0.001	
P for interaction		0.591	
**Osteoarthritis**			
Male			
T1 of total FI	381/48,975	Reference	
T2 of total FI	488/45,397	1.22 (1.06, 1.39)	0.004
T3 of total FI	648/45,286	1.43 (1.25, 1.63)	<0.001
P for trend test		<0.001	
Female			
T1 of total FI	383/44,833	Reference	
T2 of total FI	544/44,903	1.28 (1.12, 1.46)	<0.001
T3 of total FI	795/45,678	1.62 (1.43, 1.84)	<0.001
P for trend test		<0.001	
P for interaction		0.313	
**Intervertebral disc degeneration**			
Male			
T1 of total FI	57/50,159	Reference	
T2 of total FI	66/46,785	1.20 (0.84, 1.72)	0.313
T3 of total FI	112/47,331	1.93 (1.38, 2.69)	<0.001

P for trend test		<0.001	
Female			
T1 of total FI	42/46,153	Reference	
T2 of total FI	72/46,731	1.35 (0.90, 2.01)	<0.001
T3 of total FI	114/48,212	2.21 (1.53, 3.20)	<0.001
P for trend test		<0.001	
P for interaction		0.499	
**Degenerative bone and joint multimorbidity**			
Male			
T1 of total FI	18/50,304	Reference	
T2 of total FI	28/46,970	1.46 (0.80, 2.68)	0.215
T3 of total FI	51/47,554	2.32 (1.32, 4.09)	0.004
P for trend test		0.008	
Female			
T1 of total FI	41/46,190	Reference	
T2 of total FI	60/46,840	1.35 (0.90, 2.01)	0.145
T3 of total FI	110/48,286	2.21 (1.52, 3.20)	<0.001
P for trend test		<0.001	
P for interaction		0.960	

Total FI was calculated by the FI at baseline plus the FI at the final assessment. T1 was the lower tertile, T2 was the middle tertile, and T3 was the upper tertile. PYs represents the person years from first or second follow-up to DBJDs onset.

Abbreviations: PYs, person years; TDI, Townsend deprivation index; BMI, body mass index.

[a]HR and P were adjusted for age, sex, race, education, TDI, smoking status, drinking status, physical activity level and BMI.

Table S8. Association of changes in frailty status with the risk of incident degenerative bone and joint diseases and its multimorbidity stratified by age, using the "Stable non-frail group" as reference.

Variables	Cases/PYs	HR(95%CI)[a]	P[a]
**Degenerative bone and joint diseases**			
Age < 60 years			
Stable non-frail	585/69,176	Reference	
Non-frail to pre-frail/frail	332/27,977	1.36 (1.19, 1.56)	<0.001
Stable pre-frail	829/58,688	1.56 (1.40, 1.73)	<0.001
Pre-frail to non-frail	167/16,024	1.19 (1.00, 1.41)	0.052
Pre-frail to frail	108/4,548	2.47 (2.00, 3.04)	<0.001
Stable frail	63/2,924	2.09 (1.60, 2.74)	<0.001
Frail to non-frail/pre-frail	46/1,948	2.46 (1.82, 3.34)	<0.001
Age ≥ 60 years			
Stable non-frail	412/26,339	Reference	
Non-frail to pre-frail/frail	360/16,690	1.38 (1.20, 1.60)	<0.001
Stable pre-frail	812/35,319	1.40 (1.24, 1.58)	<0.001
Pre-frail to non-frail	110/5,805	1.17 (0.95, 1.44)	0.144
Pre-frail to frail	108/3,566	1.73 (1.39, 2.14)	<0.001
Stable frail	74/1,800	2.28 (1.77, 2.94)	<0.001
Frail to non-frail/pre-frail	39/1,367	1.63 (1.17, 2.27)	0.004
P for interaction		0.113	
**Osteoporosis**			
Age < 60 years			
Stable non-frail	86/71,088	Reference	
Non-frail to pre-frail/frail	52/28,990	1.60 (1.13, 2.27)	0.008
Stable pre-frail	130/61,268	1.86 (1.41, 2.45)	<0.001
Pre-frail to non-frail	26/16,599	1.24 (0.80, 1.93)	0.338
Pre-frail to frail	10/4,844	2.22 (1.15, 4.31)	0.018
Stable frail	11/3,128	4.20 (2.20, 8.03)	<0.001
Frail to non-frail/pre-frail	8/2,127	3.75 (1.80, 7.82)	<0.001
Age ≥ 60 years			
Stable non-frail	78/27,607	Reference	
Non-frail to pre-frail/frail	61/17,692	1.44 (1.02, 2.01)	0.036
Stable pre-frail	146/37,845	1.51 (1.15, 2.00)	0.004
Pre-frail to non-frail	20/6,147	1.18 (0.72, 1.93)	0.518
Pre-frail to frail	18/3,894	2.10 (1.25, 3.54)	0.005
Stable frail	12/2,074	2.95 (1.58, 5.49)	<0.001
Frail to non-frail/pre-frail	7/1,466	2.21 (1.01, 4.83)	0.047
P for interaction		0.945	
**Osteoarthritis**			
Age < 60 years			
Stable non-frail	446/69,718	Reference	
Non-frail to pre-frail/frail	262/28,265	1.38 (1.18, 1.60)	<0.001

Stable pre-frail	661/59,320	1.57 (1.39, 1.78)	<0.001
Pre-frail to non-frail	133/16,157	1.23 (1.01, 1.50)	0.036
Pre-frail to frail	83/4,624	2.27 (1.79, 2.89)	<0.001
Stable frail	49/2,973	1.90 (1.40, 2.57)	<0.001
Frail to non-frail/pre-frail	36/1,976	2.38 (1.69, 3.35)	0.002
Age ≥ 60 years			
Stable non-frail	344/26,607	Reference	
Non-frail to pre-frail/frail	298/16,864	1.34 (1.15, 1.57)	<0.001
Stable pre-frail	644/35,868	1.29 (1.13, 1.48)	<0.001
Pre-frail to non-frail	93/5,871	1.18 (0.94, 1.48)	0.161
Pre-frail to frail	91/3,626	1.63 (1.29, 2.06)	<0.001
Stable frail	64/1,822	2.18 (1.66, 2.87)	<0.001
Frail to non-frail/pre-frail	35/1,380	1.64 (1.15, 2.33)	0.006
P for interaction		0.090	
**Intervertebral disc degeneration**			
Age < 60 years			
Stable non-frail	83/71,129	Reference	
Non-frail to pre-frail/frail	37/29,015	1.07 (0.72, 1.58)	0.732
Stable pre-frail	108/61,312	1.45 (1.08, 1.94)	0.014
Pre-frail to non-frail	18/16,604	0.92 (0.55, 1.54)	0.753
Pre-frail to frail	22/4,817	3.49 (2.15, 5.67)	<0.001
Stable frail	9/3,128	2.05 (1.01, 4.19)	0.047
Frail to non-frail/pre-frail	7/2,129	2.54 (1.16, 5.55)	0.020
Age ≥ 60 years			
Stable non-frail	27/27,775	Reference	
Non-frail to pre-frail/frail	28/17,791	1.62 (0.95, 2.76)	0.078
Stable pre-frail	91/38,036	2.37 (1.53, 3.67)	<0.001
Pre-frail to non-frail	10/6,175	1.63 (0.78, 3.39)	0.189
Pre-frail to frail	13/3,894	3.19 (1.62, 6.28)	<0.001
Stable frail	8/2,090	3.52 (1.56, 7.95)	0.003
Frail to non-frail/pre-frail	2/1,475	1.27 (0.30, 5.42)	0.749
P for interaction		0.283	
**Degenerative bone and joint multimorbidity**			
Age < 60 years			
Stable non-frail	29/71,338	Reference	
Non-frail to pre-frail/frail	17/29,124	1.49 (0.81, 2.73)	0.198
Stable pre-frail	68/61,517	2.50 (1.60, 3.90)	<0.001
Pre-frail to non-frail	10/16,657	1.38 (0.67, 2.86)	0.378
Pre-frail to frail	7/4,860	3.27 (1.40, 7.64)	0.006
Stable frail	6/3,147	3.58 (1.42, 9.00)	0.007
Frail to non-frail/pre-frail	5/2,132	4.77 (1.80, 12.60)	0.002
Age ≥ 60 years			
Stable non-frail	35/27,781	Reference	
Non-frail to pre-frail/frail	26/17,800	1.34 (0.80, 2.25)	0.257

Stable pre-frail	64/38,143	1.38 (0.90, 2.09)	0.134
Pre-frail to non-frail	12/6,182	1.53 (0.79, 2.97)	0.206
Pre-frail to frail	14/3,910	3.15 (1.66, 5.96)	<0.001
Stable frail	10/2,081	4.41 (2.12, 9.16)	<0.001
Frail to non-frail/pre-frail	5/1,473	2.90 (1.12, 7.55)	0.029
P for interaction		0.304	

PYs represents the person years from first or second follow-up to DBJDs onset.

Abbreviations: PYs, person years; TDI, Townsend deprivation index; BMI, body mass index.

[a]HR and P were adjusted for age, sex, race, education, TDI, smoking status, drinking status, physical activity level and BMI.

Table S9. Association of changes in frailty status with the risk of incident degenerative bone and joint diseases and its multimorbidity stratified by age, using each stable frailty status group as its own reference.

Variables	Cases/PYs	HR(95%CI)[a]	P[a]
**Degenerative bone and joint diseases**			
Age < 60 years			
Stable non-frail	585/69,176	Reference	
Non-frail to pre-frail/frail	332/27,977	1.36 (1.18, 1.56)	<0.001
Age ≥ 60 years			
Stable non-frail	412/26,339	Reference	
Non-frail to pre-frail/frail	360/16,690	1.38 (1.20, 1.60)	<0.001
P for interaction		0.865	
Age < 60 years			
Stable pre-frail	829/58,688	Reference	
Pre-frail to non-frail	167/16,024	0.76 (0.64, 0.90)	0.001
Pre-frail to frail	108/4,548	1.59 (1.30, 1.95)	<0.001
Age ≥ 60 years			
Stable pre-frail	812/35,319	Reference	
Pre-frail to non-frail	110/5,805	0.83 (0.68, 1.02)	0.074
Pre-frail to frail	108/3,566	1.22 (1.00, 1.50)	0.052
P for interaction		0.152	
Age < 60 years			
Stable frail	63/2,924	Reference	
Frail to non-frail/pre-frail	46/1,948	1.18 (0.79, 1.75)	0.408
Age ≥ 60 years			
Stable frail	74/1,800	Reference	
Frail to non-frail/pre-frail	39/1,367	0.70 (0.46, 1.07)	0.101
P for interaction		0.129	
**Osteoporosis**			
Age < 60 years			
Stable non-frail	86/71,088	Reference	
Non-frail to pre-frail/frail	52/28,990	1.58 (1.11, 2.24)	0.011
Age ≥ 60 years			
Stable non-frail	78/27,607	Reference	
Non-frail to pre-frail/frail	61/17,692	1.46 (1.04, 2.06)	0.030
P for interaction		0.682	
Age < 60 years			
Stable pre-frail	130/61,268	Reference	
Pre-frail to non-frail	26/16,599	0.68 (0.44, 1.04)	0.071
Pre-frail to frail	10/4,844	1.17 (0.61, 2.25)	0.634
Age ≥ 60 years			
Stable pre-frail	146/37,845	Reference	
Pre-frail to non-frail	20/6,147	0.78 (0.49, 1.25)	0.299
Pre-frail to frail	18/3,894	1.33 (0.81, 2.19)	0.258

P for interaction		0.873	
Age < 60 years			
Stable frail	63/2,924	Reference	
Frail to non-frail/pre-frail	46/1,948	0.67 (0.18, 2.48)	0.453
Age ≥ 60 years			
Stable frail	74/1,800	Reference	
Frail to non-frail/pre-frail	39/1,367	0.85 (0.08, 9.41)	0.790
P for interaction		0.717	
**Osteoarthritis**			
Age < 60 years			
Stable non-frail	446/69,718	Reference	
Non-frail to pre-frail/frail	262/28,265	1.37 (1.17, 1.59)	<0.001
Age ≥ 60 years			
Stable non-frail	344/26,607	Reference	
Non-frail to pre-frail/frail	298/16,864	1.34 (1.15, 1.57)	<0.001
P for interaction		0.880	
Age < 60 years			
Stable pre-frail	133/16,157	Reference	
Pre-frail to non-frail	83/4,624	0.77 (0.64, 0.93)	0.008
Pre-frail to frail	49/2,973	1.48 (1.17, 1.86)	<0.001
Age ≥ 60 years			
Stable pre-frail	644/35,868	Reference	
Pre-frail to non-frail	93/5,871	0.91 (0.73, 1.13)	0.398
Pre-frail to frail	91/3,626	1.25 (1.00, 1.57)	0.047
P for interaction		0.358	
Age < 60 years			
Stable frail	49/2,973	Reference	
Frail to non-frail/pre-frail	36/1,976	1.30 (0.83, 2.05)	0.248
Age ≥ 60 years			
Stable frail	64/1,822	Reference	
Frail to non-frail/pre-frail	35/1,380	0.75 (0.48, 1.19)	0.218
P for interaction		0.141	
**Intervertebral disc degeneration**			
Age < 60 years			
Stable non-frail	83/71,129	Reference	
Non-frail to pre-frail/frail	37/29,015	1.10 (0.74, 1.64)	0.626
Age ≥ 60 years			
Stable non-frail	27/27,775	Reference	
Non-frail to pre-frail/frail	28/17,791	1.56 (0.90, 2.72)	0.110
P for interaction		0.306	
Age < 60 years			
Stable pre-frail	108/61,312	Reference	
Pre-frail to non-frail	18/16,604	0.64 (0.39, 1.07)	0.087
Pre-frail to frail	22/4,817	2.32 (1.45, 3.71)	<0.001

Age ≥ 60 years			
Stable pre-frail	91/38,036	Reference	
Pre-frail to non-frail	10/6,175	0.69 (0.26, 1.33)	0.264
Pre-frail to frail	13/3,894	1.36 (0.31, 2.46)	0.311
P for interaction		0.266	
Age < 60 years			
Stable frail	9/3,128	Reference	
Frail to non-frail/pre-frail	7/2,129	1.29 (0.14, 11.91)	0.674
Age ≥ 60 years			
Stable frail	8/2,090	Reference	
Frail to non-frail/pre-frail	2/1,475	0.53 (0.11, 2.50)	0.423
P for interaction		0.288	
**Degenerative bone and joint multimorbidity**			
Age < 60 years			
Stable non-frail	29/71,338	Reference	
Non-frail to pre-frail/frail	17/29,124	1.51 (0.81, 2.84)	0.190
Age ≥ 60 years			
Stable non-frail	35/27,781	Reference	
Non-frail to pre-frail/frail	26/17,800	1.38 (0.81, 2.34)	0.072
P for interaction		0.769	
Age < 60 years			
Stable pre-frail	68/61,517	Reference	
Pre-frail to non-frail	10/16,657	0.57 (0.29, 1.12)	0.103
Pre-frail to frail	7/4,860	1.27 (0.57, 2.83)	0.547
Age ≥ 60 years			
Stable pre-frail	64/38,143	Reference	
Pre-frail to non-frail	12/6,182	1.12 (0.60, 2.10)	0.724
Pre-frail to frail	14/3,910	2.20 (1.21, 3.99)	0.011
P for interaction		0.243	
Age < 60 years			
Stable frail	6/3,147	Reference	
Frail to non-frail/pre-frail	5/2,132	1.37 (0.40, 4.70)	0.613
Age ≥ 60 years			
Stable frail	10/2,081	Reference	
Frail to non-frail/pre-frail	5/1,473	0.98 (0.29, 3.36)	0.974
P for interaction		0.451	

PYs represents the person years from first or second follow-up to DBJDs onset.

Abbreviations: PYs, person years; TDI, Townsend deprivation index; BMI, body mass index.

[a]HR and P were adjusted for age, sex, race, education, TDI, smoking status, drinking status, physical activity level and BMI.

Table S10. Association the rate of change in frailty index with incident degenerative bone and joint diseases and its multimorbidity stratified by age.

Variables	Cases/PYs	HR(95%CI)[a]	P[a]
**Degenerative bone and joint diseases**			
Age < 60 years			
T1 of △FI / year	755/67,822	Reference	
T2 of △FI / year	559/54,064	1.11 (1.00, 1.25)	0.058
T3 of △FI / year	816/59,399	1.41 (1.27, 1.56)	<0.001
P for trend test		<0.001	
Age ≥ 60 years			
T1 of △FI / year	556/28,468	Reference	
T2 of △FI / year	527/24,999	1.27 (1.12, 1.43)	<0.001
T3 of △FI / year	832/37,419	1.32 (1.18, 1.48)	<0.001
P for trend test		<0.001	
P for interaction		0.055	
**Osteoporosis**			
Age < 60 years			
T1 of △FI / year	120/70,444	Reference	
T2 of △FI / year	84/55,574	1.21 (0.91, 1.62)	0.195
T3 of △FI / year	119/62,026	1.52 (1.17, 1.98)	0.002
P for trend test		0.002	
Age ≥ 60 years			
T1 of △FI / year	93/30,262	Reference	
T2 of △FI / year	89/26,515	1.40 (1.04, 1.89)	0.028
T3 of △FI / year	160/39,948	1.72 (1.32, 2.25)	<0.001
P for trend test		<0.001	
P for interaction		0.472	
**Osteoarthritis**			
Age < 60 years			
T1 of △FI / year	594/68,423	Reference	
T2 of △FI / year	444/54,450	1.12 (0.98, 1.27)	0.091
T3 of △FI / year	632/60,162	1.35 (1.20, 1.51)	<0.001
P for trend test		<0.001	
Age ≥ 60 years			
T1 of △FI / year	462/28,813	Reference	
T2 of △FI / year	440/25,243	1.26 (1.10, 1.44)	<0.001
T3 of △FI / year	667/37,982	1.24 (1.10, 1.41)	<0.001
P for trend test		0.001	
P for interaction		0.081	
**Intervertebral disc degeneration**			
Age < 60 years			
T1 of △FI / year	106/70,465	Reference	
T2 of △FI / year	67/55,640	0.98 (0.72, 1.35)	0.915
T3 of △FI / year	111/62,029	1.41 (1.07, 1.86)	0.016

P for trend test		0.016	
Age ≥ 60 years			
T1 of △FI / year	55/30,355	Reference	
T2 of △FI / year	50/26,646	1.42 (0.85, 2.36)	0.179
T3 of △FI / year	74/40,236	1.54 (0.98, 2.42)	0.059
P for trend test		0.063	
P for interaction		0.306	
**Degenerative bone and joint multimorbidity**			
Age < 60 years			
T1 of △FI / year	63/70,663	Reference	
T2 of △FI / year	35/55,762	1.09 (0.71, 1.68)	0.678
T3 of △FI / year	44/62,350	1.05 (0.70, 1.56)	0.822
P for trend test		0.805	
Age ≥ 60 years			
T1 of △FI / year	53/30,424	Reference	
T2 of △FI / year	47/26,651	1.29 (0.87, 1.92)	0.206
T3 of △FI / year	66/40,295	1.27 (0.88, 1.83)	0.207
P for trend test		0.211	
P for interaction		0.425	

△FI was calculated by the FI at the final assessment minus the FI at baseline. T1 was the lower tertile, T2 was the middle tertile, and T3 was the upper tertile. PYs represents the person years from first or second follow-up to DBJDs onset.

Abbreviations: PYs, person years; TDI, Townsend deprivation index; BMI, body mass index.

[a]HR and P were adjusted for age, sex, race, education, TDI, smoking status, drinking status, physical activity level, BMI and baseline FI.

Table S11. Association of total frailty index with incident degenerative bone and joint diseases and its multimorbidity stratified by age.

Variables	Cases/PYs	HR(95%CI)[a]	P[a]
**Degenerative bone and joint diseases**			
Age < 60 years			
T1 of total FI	560/67,764	Reference	
T2 of total FI	670/58,698	1.33 (1.19, 1.49)	<0.001
T3 of total FI	900/54,823	1.81 (1.62, 2.01)	<0.001
P for trend test		<0.001	
Age ≥ 60 years			
T1 of total FI	392/25,307	Reference	
T2 of total FI	623/30,635	1.28 (1.12, 1.45)	<0.001
T3 of total FI	900/34,944	1.54 (1.37, 1.74)	<0.001
P for trend test		<0.001	
P for interaction		0.136	
**Osteoporosis**			
Age < 60 years			
T1 of total FI	77/69,582	Reference	
T2 of total FI	105/60,846	1.59 (1.18, 2.13)	0.002
T3 of total FI	141/57,616	2.44 (1.83, 3.24)	<0.001
P for trend test		<0.001	
Age ≥ 60 years			
T1 of total FI	74/26,523	Reference	
T2 of total FI	112/32,481	1.32 (0.98, 1.78)	0.065
T3 of total FI	156/37,721	1.72 (1.30, 2.28)	<0.001
P for trend test		<0.001	
P for interaction		0.261	
**Osteoarthritis**			
Age < 60 years			
T1 of total FI	435/68,253	Reference	
T2 of total FI	527/59,282	1.32 (1.16, 1.50)	<0.001
T3 of total FI	708/55,499	1.74 (1.54, 1.97)	<0.001
P for trend test		<0.001	
Age ≥ 60 years			
T1 of total FI	329/25,555	Reference	
T2 of total FI	505/31,019	1.21 (1.06, 1.40)	0.006
T3 of total FI	735/35,464	1.44 (1.26, 1.64)	<0.001
P for trend test		<0.001	
P for interaction		0.111	
**Intervertebral disc degeneration**			
Age < 60 years			
T1 of total FI	77/69,618	Reference	
T2 of total FI	76/60,885	1.11 (0.80, 1.52)	0.536
T3 of total FI	131/57,630	1.91 (1.43, 2.56)	<0.001

P for trend test		<0.001	
Age ≥ 60 years			
T1 of total FI	22/26,693	Reference	
T2 of total FI	62/32,631	2.27 (1.39, 3.71)	0.001
T3 of total FI	95/37,913	2.88 (1.79, 4.63)	<0.001
P for trend test		<0.001	
P for interaction		0.048	
**Degenerative bone and joint multimorbidity**			
Age < 60 years			
T1 of total FI	28/69,799	Reference	
T2 of total FI	36/61,123	1.41 (0.86, 2.33)	0.176
T3 of total FI	78/57,854	2.98 (1.91, 4.67)	<0.001
P for trend test		<0.001	
Age ≥ 60 years			
T1 of total FI	31/26,696	Reference	
T2 of total FI	52/32,688	1.41 (0.90, 2.21)	0.136
T3 of total FI	83/37,987	1.95 (1.28, 2.98)	0.002
P for trend test		0.002	
P for interaction		0.103	

Total FI was calculated by the FI at baseline plus the FI at the final assessment. T1 was the lower tertile, T2 was the middle tertile, and T3 was the upper tertile. PYs represents the person years from first or second follow-up to DBJDs onset.

Abbreviations: PYs, person years; TDI, Townsend deprivation index; BMI, body mass index.

[a]HR and P were adjusted for age, sex, race, education, TDI, smoking status, drinking status, physical activity level and BMI.

Table S12. Association of changes in frailty status with the risk of incident degenerative bone and joint diseases and its multimorbidity when using the cut-off value 1.

Variables	Cases/PYs	HR(95%CI)[a]	P[a]	HR(95%CI)[a]	P[a]
**Degenerative bone and joint diseases**					
Stable non-frail	854/85,061	Reference		Reference	
Non-frail to pre-frail/frail	835/55,121	1.34 (1.22, 1.48)	<0.001	1.33 (1.21, 1.46)	<0.001
Stable pre-frail	1,641/94,007	1.47 (1.35, 1.60)	<0.001	Reference	
Pre-frail to non-frail	197/16,638	1.14 (0.97, 1.33)	0.104	0.77 (0.66, 0.89)	<0.001
Pre-frail to frail	296/13,306	1.78 (1.56, 2.04)	<0.001	1.22 (1.07, 1.38)	0.002
Stable frail	137/4,762	2.11 (1.75, 2.54)	<0.001	Reference	
Frail to non-frail/pre-frail	85/3,277	1.95 (1.56, 2.44)	<0.001	0.91 (0.69, 1.20)	0.512
**Osteoporosis**					
Stable non-frail	137/87,696	Reference		Reference	
Non-frail to pre-frail/frail	140/57,680	1.45 (1.15, 1.84)	0.002	1.47 (1.16, 1.87)	0.002
Stable pre-frail	276/99,113	1.65 (1.34, 2.03)	<0.001	Reference	
Pre-frail to non-frail	26/17,221	0.99 (0.65, 1.51)	0.956	0.61 (0.41, 0.91)	0.017
Pre-frail to frail	48/14,263	1.97 (1.41, 2.74)	<0.001	1.18 (0.87, 1.61)	0.294
Stable frail	23/5,240	3.38 (2.15, 5.31)	<0.001	Reference	
Frail to non-frail/pre-frail	15/3,555	2.75 (1.60, 4.70)	<0.001	0.77 (0.38, 1.56)	0.448
**Osteoarthritis**					
Stable non-frail	680/85,706	Reference		Reference	
Non-frail to pre-frail/frail	670/55,748	1.32 (1.18, 1.47)	<0.001	1.29 (1.16, 1.44)	<0.001
Stable pre-frail	1,305/95,188	1.41 (1.28, 1.55)	<0.001	Reference	
Pre-frail to non-frail	162/16,754	1.17 (0.98, 1.39)	0.076	0.81 (0.69, 0.96)	0.014
Pre-frail to frail	238/13,525	1.69 (1.45, 1.96)	<0.001	1.21 (1.05, 1.39)	0.008
Stable frail	113/4,833	1.96 (1.59, 2.40)	<0.001	Reference	
Frail to non-frail /pre-frail	71/3,317	1.89 (1.48, 2.42)	<0.001	0.97 (0.71, 1.31)	0.820
**Intervertebral disc degeneration**					
Stable non-frail	94/87,842	Reference		Reference	
Non-frail to pre-frail/frail	81/57,869	1.25 (0.93, 1.69)	0.140	1.29 (0.95, 1.74)	0.104
Stable pre-frail	199/99,348	1.75 (1.36, 2.24)	<0.001	Reference	
Pre-frail to non-frail	22/17,222	1.18 (0.74, 1.88)	0.493	0.68 (0.44, 1.07)	0.093
Pre-frail to frail	41/14,268	2.42 (1.66, 3.51)	0.025	1.37 (0.98, 1.93)	0.067
Stable frail	17/5,256	2.49 (1.46, 4.24)	<0.001	Reference	
Frail to non-frail/pre-frail	9/3,566	2.03 (1.02, 4.06)	0.045	0.78 (0.31, 1.98)	0.568
**Degenerative bone and joint multimorbidity**					
Stable non-frail	54/88,023	Reference		Reference	
Non-frail to pre-frail/frail	53/58,019	1.29 (0.88, 1.88)	0.198	1.29 (0.88, 1.91)	0.193
Stable pre-frail	132/99,660	1.74 (1.26, 2.40)	<0.001	Reference	
Pre-frail to non-frail	13/17,268	1.25 (0.68, 2.31)	0.465	0.72 (0.40, 1.28)	0.257
Pre-frail to frail	30/14,342	2.48 (1.57, 3.91)	<0.001	1.41 (0.94, 2.10)	0.096
Stable frail	16/5,266	3.72 (2.08, 6.65)	<0.001	Reference	
Frail to non-frail /pre-frail	10/3,567	3.33 (1.67, 6.61)	<0.001	0.97 (0.39, 2.42)	0.938

In the cut-off value 1, non-frail was defined as FI ≤0.08, pre-frail was defined as 0.08< FI ≤0.25, frail was defined as FI >0.25. PYs represents the person years from first or second follow-up to DBJDs onset.

Abbreviations: PYs, person years; TDI, Townsend deprivation index; BMI, body mass index.

[a]HR and P were adjusted for age, sex, race, education, TDI, smoking status, drinking status, physical activity level and BMI.

Table S13. Association of changes in frailty status with the risk of incident degenerative bone and joint diseases and its multimorbidity when using the cut-off value 2.

Variables	Cases/PYs	HR(95%CI)[a]	P[a]	HR(95%CI)[a]	P[a]
**Degenerative bone and joint diseases**					
Stable non-frail	997/95,515	Reference		Reference	
Non-frail to pre-frail/frail	692/44,667	1.34 (1.22, 1.48)	<0.001	1.33 (1.21, 1.47)	<0.001
Stable pre-frail	1,222/74,612	1.37 (1.25, 1.49)	<0.001	Reference	
Pre-frail to non-frail	271/21,521	1.15 (1.01, 1.32)	0.037	0.84 (0.74, 0.96)	0.011
Pre-frail to frail	371/15,549	1.86 (1.65, 2.10)	<0.001	1.36 (1.21, 1.53)	<0.001
Stable frail	342/13,060	1.88 (1.66, 2.14)	<0.001	Reference	
Frail to non-frail/pre-frail	150/7,248	1.68 (1.41, 2.00)	<0.001	0.87 (0.71, 1.06)	0.161
**Osteoporosis**					
Stable non-frail	164/98,695	Reference		Reference	
Non-frail to pre-frail/frail	113/46,682	1.46 (1.15, 1.86)	0.002	1.46 (1.14, 1.87)	0.003
Stable pre-frail	206/78,455	1.48 (1.20, 1.82)	<0.001	Reference	
Pre-frail to non-frail	43/22,418	1.12 (0.80, 1.47)	0.496	0.77 (0.55, 1.07)	0.115
Pre-frail to frail	58/16,590	2.15 (1.59, 2.92)	<0.001	1.42 (1.06, 1.91)	0.019
Stable frail	52/14,217	2.42 (1.75, 3.33)	<0.001	Reference	
Frail to non-frail/pre-frail	29/7,713	2.32 (1.56, 3.45)	<0.001	0.93 (0.58, 1.50)	0.771
**Osteoarthritis**					
Stable non-frail	790/96,326	Reference		Reference	
Non-frail to pre-frail/frail	560/45,129	1.34 (1.20, 1.49)	<0.001	1.31 (1.18, 1.47)	<0.001
Stable pre-frail	972/75,483	1.33 (1.21, 1.46)	<0.001	Reference	
Pre-frail to non-frail	221/21,717	1.18 (1.02, 1.37)	0.030	0.88 (0.76, 1.02)	0.091
Pre-frail to frail	288/15,842	1.69 (1.48, 1.94)	<0.001	1.28 (1.12, 1.46)	<0.001
Stable frail	284/13,232	1.80 (1.56, 2.08)	<0.001	Reference	
Frail to non-frail/pre-frail	124/7,344	1.65 (1.37, 2.00)	<0.001	0.89 (0.72, 1.11)	0.302
**Intervertebral disc degeneration**					
Stable non-frail	110/98,904	Reference		Reference	
Non-frail to pre-frail/frail	65/46,806	1.21 (0.89, 1.65)	0.222	1.24 (0.90, 1.69)	0.185
Stable pre-frail	137/78,661	1.49 (1.16, 1.93)	0.002	Reference	
Pre-frail to non-frail	28/22,448	1.10 (0.73, 1.68)	0.638	0.75 (0.50, 1.13)	0.167
Pre-frail to frail	58/16,599	2.86 (2.06, 3.97)	<0.001	1.89 (1.38, 2.58)	<0.001
Stable frail	41/14,227	2.26 (1.55, 3.28)	<0.001	Reference	
Frail to non-frail/pre-frail	24/7,726	2.59 (1.65, 4.05)	<0.001	1.16 (0.68, 1.96)	0.586
**Degenerative bone and joint multimorbidity**					
Stable non-frail	64/99,119	Reference		Reference	
Non-frail to pre-frail/frail	43/46,924	1.38 (0.93, 2.03)	0.108	1.37 (0.92, 2.04)	0.118
Stable pre-frail	89/78,879	1.49 (1.08, 2.07)	0.016	Reference	
Pre-frail to non-frail	20/22,509	1.31 (0.79, 2.17)	0.295	0.88 (0.54, 1.44)	0.611
Pre-frail to frail	31/16,703	2.45 (1.58, 3.79)	<0.001	1.58 (1.04, 2.40)	0.031
Stable frail	35/14,271	2.96 (1.92, 4.56)	<0.001	Reference	
Frail to non-frail/pre-frail	26/7,740	4.47 (2.81, 7.11)	<0.001	1.45 (0.85, 2.48)	0.168

In the cut-off value 2, non-frail was defined as FI ≤0.10, pre-frail was defined as 0.10< FI ≤0.21, frail was defined as FI >0.21. PYs represents the person years from first or second follow-up to DBJDs onset.

Abbreviations: PYs, person years; TDI, Townsend deprivation index; BMI, body mass index.

[a]HR and P were adjusted for age, sex, race, education, TDI, smoking status, drinking status, physical activity level and BMI.

Table S14. Association of changes in frailty status with the risk of incident degenerative bone and joint diseases and its multimorbidity excluding cases occurring within the first year of follow-up.

Variables	Cases/PYs	HR(95%CI)[a]	P[a]	HR(95%CI)[a]	P[a]
**Degenerative bone and joint diseases**					
Stable non-frail	870/95,455	Reference		Reference	
Non-frail to pre-frail/frail	578/44,611	1.32 (1.18, 1.46)	<0.001	1.30 (1.17, 1.45)	<0.001
Stable pre-frail	1,390/93,879	1.39 (1.28, 1.52)	<0.001	Reference	
Pre-frail to non-frail	232/21,809	1.12 (0.97, 1.29)	0.127	0.79 (0.69, 0.91)	0.001
Pre-frail to frail	180/8,099	1.92 (1.63, 2.26)	<0.001	1.40 (1.19, 1.63)	<0.001
Stable frail	113/4,712	1.96 (1.60, 2.39)	<0.001	Reference	
Frail to non-frail/pre-frail	68/3,306	1.73 (1.35, 2.22)	<0.001	0.89 (0.65, 1.21)	0.457
**Osteoporosis**					
Stable non-frail	144/98,687	Reference		Reference	
Non-frail to pre-frail/frail	108/46,681	1.63 (1.27, 2.10)	<0.001	1.62 (1.26, 2.09)	<0.001
Stable pre-frail	246/99,097	1.62 (1.31, 2.00)	<0.001	Reference	
Pre-frail to non-frail	44/22,746	1.29 (0.92, 1.81)	0.138	0.80 (0.58, 1.11)	0.185
Pre-frail to frail	22/8,735	1.87 (1.19, 2.94)	0.007	1.14 (0.73, 1.77)	0.567
Stable frail	21/5,201	3.39 (2.12, 5.43)	<0.001	Reference	
Frail to non-frail/pre-frail	13/3,592	2.61 (1.47, 4.63)	0.001	0.70 (0.33, 1.51)	0.348
**Osteoarthritis**					
Stable non-frail	695/96,281	Reference		Reference	
Non-frail to pre-frail/frail	465/45,083	1.29 (1.14, 1.45)	<0.001	1.27 (1.12, 1.43)	<0.001
Stable pre-frail	1,106/95,087	1.33 (1.21, 1.47)	<0.001	Reference	
Pre-frail to non-frail	190/22,011	1.14 (0.97, 1.34)	0.106	0.84 (0.72, 0.98)	0.026
Pre-frail to frail	147/8,240	1.79 (1.50, 2.15)	<0.001	1.37 (1.15, 1.64)	<0.001
Stable frail	91/4,784	1.77 (1.41, 2.21)	<0.001	Reference	
Frail to non-frail/pre-frail	57/3,348	1.68 (1.28, 2.21)	<0.001	0.96 (0.71, 1.30)	0.790
**Intervertebral disc degeneration**					
Stable non-frail	96/98,897	Reference		Reference	
Non-frail to pre-frail/frail	49/46,796	1.05 (0.75, 1.49)	0.763	1.08 (0.76, 1.53)	0.682
Stable pre-frail	173/99,336	1.67 (1.29, 2.15)	<0.001	Reference	
Pre-frail to non-frail	21/22,775	0.93 (0.58, 1.49)	0.763	0.56 (0.36, 0.89)	0.014
Pre-frail to frail	30/8,709	3.12 (2.05, 4.76)	<0.001	1.83 (1.23, 2.71)	0.003
Stable frail	17/5,218	2.72 (1.59, 4.64)	<0.001	Reference	
Frail to non-frail/pre-frail	7/3,603	1.69 (0.78, 3.68)	0.184	0.60 (0.21, 1.71)	0.300
**Degenerative bone and joint multimorbidity**					
Stable non-frail	62/99,118	Reference		Reference	
Non-frail to pre-frail/frail	41/46,922	1.36 (0.91, 2.03)	0.128	1.38 (0.92, 2.06)	0.117
Stable pre-frail	128/99,658	1.74 (1.28, 2.37)	<0.001	Reference	
Pre-frail to non-frail	22/22,840	1.47 (0.90, 2.39)	0.124	0.86 (0.54, 1.36)	0.518
Pre-frail to frail	19/8,771	2.85 (1.68, 4.83)	<0.001	1.75 (1.09, 2.81)	0.020
Stable frail	16/5,228	3.91 (2.20, 6.93)	<0.001	Reference	
Frail to non-frail/pre-frail	9/3,604	3.08 (1.51, 6.28)	0.002	0.86 (0.33, 2.24)	0.728

In the cut-off value 1, non-frail was defined as FI ≤0.08, pre-frail was defined as 0.08< FI ≤0.25, frail was defined as FI >0.25. PYs represents the person years from first or second follow-up to DBJDs onset.

Abbreviations: PYs, person years; TDI, Townsend deprivation index; BMI, body mass index.

[a]HR and P were adjusted for age, sex, race, education, TDI, smoking status, drinking status, physical activity level and BMI.

Table S15. Association the rate of change in frailty index with incident degenerative bone and joint diseases and its multimorbidity excluding cases occurring within the first year of follow-up.

Variables	Cases/PYs	HR(95%CI)[a]	P[a]
**Degenerative bone and joint diseases**			
T1 of △FI / year	1,120/96,199	Reference	
T2 of △FI / year	905/78,971	1.17 (1.07, 1.28)	<0.001
T3 of △FI / year	1,406/96,700	1.33 (1.22, 1.44)	<0.001
P for trend test		<0.001	
△FI per 0.01-point/year increase		1.11 (1.07, 1.15)	<0.001
△FI per 0.05-point/year increase		1.69 (1.43, 2.01)	<0.001
**Osteoporosis**			
T1 of △FI / year	191/100,695	Reference	
T2 of △FI / year	153/82,079	1.30 (1.04, 1.61)	0.021
T3 of △FI / year	254/101,964	1.59 (1.31, 1.94)	<0.001
P for trend test		<0.001	
△FI per 0.01-point/year increase		1.14 (1.06, 1.24)	0.001
△FI per 0.05-point/year increase		1.96 (1.31, 2.93)	0.001
**Osteoarthritis**			
T1 of △FI / year	909/97,166	Reference	
T2 of △FI / year	739/79,619	1.16 (1.05, 1.28)	0.003
T3 of △FI / year	1,103/98,047	1.23 (1.12, 1.35)	<0.001
P for trend test		<0.001	
△FI per 0.01-point/year increase		1.08 (1.04, 1.12)	<0.001
△FI per 0.05-point/year increase		1.44 (1.19, 1.75)	<0.001
**Intervertebral disc degeneration**			
T1 of △FI / year	137/100,807	Reference	
T2 of △FI / year	96/82,276	1.10 (0.84, 1.45)	0.467
T3 of △FI / year	160/102,252	1.37 (1.08, 1.74)	0.009
P for trend test		0.009	
△FI per 0.01-point/year increase		1.22 (1.11, 1.35)	<0.001
△FI per 0.05-point/year increase		2.75 (1.69, 4.48)	<0.001
**Degenerative bone and joint multimorbidity**			
T1 of △FI / year	114/101,087	Reference	
T2 of △FI / year	77/82,412	1.20 (0.89, 1.61)	0.235
T3 of △FI / year	106/102,642	1.11 (0.84, 1.46)	0.456
P for trend test		0.455	
△FI per 0.01-point/year increase		1.05 (0.94, 1.17)	0.368
△FI per 0.05-point/year increase		1.28 (0.75, 2.21)	0.368

△FI was calculated by the FI at the final assessment minus the FI at baseline. T1 was the lower tertile, T2 was the middle tertile, and T3 was the upper tertile. PYs represents the person years from first or second follow-up to DBJDs onset.

Abbreviations: PYs, person years; TDI, Townsend deprivation index; BMI, body mass index.

[a]HR and P were adjusted for age, sex, race, education, TDI, smoking status, drinking status, physical activity level, BMI and baseline FI.

Table S16. Association of total frailty index with incident degenerative bone and joint diseases and its multimorbidity excluding cases occurring within the first year of follow-up.

Variables	Cases/PYs	HR(95%CI)[a]	P[a]
**Degenerative bone and joint diseases**			
T1 of total FI	825/93,013	Reference	
T2 of total FI	1,094/89,231	1.26 (1.15, 1.38)	<0.001
T3 of total FI	1,512/89,626	1.57 (1.44, 1.71)	<0.001
P for trend test		<0.001	
Per 0.01-point increase		1.02 (1.01, 1.02)	<0.001
Per 0.05-point increase		1.08 (1.07, 1.09)	<0.001
**Osteoporosis**			
T1 of total FI	134/96,099	Reference	
T2 of total FI	202/93,321	1.49 (1.19, 1.85)	<0.001
T3 of total FI	262/95,318	1.92 (1.56, 2.38)	<0.001
P for trend test		<0.001	
Per 0.01-point increase		1.02 (1.02, 1.03)	<0.001
Per 0.05-point increase		1.13 (1.09, 1.16)	<0.001
**Osteoarthritis**			
T1 of total FI	663/93,763	Reference	
T2 of total FI	882/90,224	1.23 (1.11, 1.36)	<0.001
T3 of total FI	1,206/90,847	1.47 (1.33, 1.62)	<0.001
P for trend test		<0.001	
Per 0.01-point increase		1.01 (1.01, 1.02)	<0.001
Per 0.05-point increase		1.07 (1.05, 1.08)	<0.001
**Intervertebral disc degeneration**			
T1 of total FI	87/96,304	Reference	
T2 of total FI	103/93,496	1.17 (0.88, 1.57)	0.273
T3 of total FI	203/95,534	2.12 (1.63, 2.75)	<0.001
P for trend test		<0.001	
Per 0.01-point increase		1.03 (1.02, 1.03)	<0.001
Per 0.05-point increase		1.15 (1.11, 1.19)	<0.001
**Degenerative bone and joint multimorbidity**			
T1 of total FI	56/96,493	Reference	
T2 of total FI	87/93,810	1.74 (1.15, 2.63)	0.009
T3 of total FI	154/95,838	2.57 (1.73, 3.82)	<0.001
P for trend test		<0.001	
Per 0.01-point increase		1.03 (1.02, 1.04)	<0.001
Per 0.05-point increase		1.17 (1.12, 1.22)	<0.001

Total FI was calculated by the FI at baseline plus the FI at the final assessment. T1 was the lower tertile, T2 was the middle tertile, and T3 was the upper tertile. PYs represents the person years from first or second follow-up to DBJDs onset.

Abbreviations: PYs, person years; TDI, Townsend deprivation index; BMI, body mass index.
[a]HR and P were adjusted for age, sex, race, education, TDI, smoking status, drinking status, physical activity level and BMI.

Table S17. Association of changes in frailty status with the risk of incident degenerative bone and joint diseases and its multimorbidity excluding cases occurring within the first two years of follow-up.

Variables	Cases/PYs	HR(95%CI)[a]	P[a]	HR(95%CI)[a]	P[a]
**Degenerative bone and joint diseases**					
Stable non-frail	711/95,218	Reference		Reference	
Non-frail to pre-frail/frail	473/44,455	1.36 (1.21, 1.53)	<0.001	1.34 (1.19, 1.51)	<0.001
Stable pre-frail	1,147/93,513	1.41 (1.29, 1.56)	<0.001	Reference	
Pre-frail to non-frail	181/21,728	1.08 (0.91, 1.27)	0.374	0.75 (0.64, 0.88)	<0.001
Pre-frail to frail	144/8,043	1.94 (1.62, 2.33)	<0.001	1.39 (1.17, 1.66)	<0.001
Stable frail	97/4,686	2.08 (1.68, 2.59)	<0.001	Reference	
Frail to non-frail/pre-frail	51/3,281	1.59 (1.20, 2.12)	0.001	0.77 (0.54, 1.09)	0.142
**Osteoporosis**					
Stable non-frail	122/98,655	Reference		Reference	
Non-frail to pre-frail/frail	87/46,649	1.61 (1.22, 2.12)	<0.001	1.60 (1.21, 2.11)	0.001
Stable pre-frail	214/99,048	1.67 (1.34, 2.10)	<0.001	Reference	
Pre-frail to non-frail	37/22,734	1.30 (0.90, 1.88)	0.166	0.78 (0.55, 1.11)	0.166
Pre-frail to frail	20/8,732	2.06 (1.27, 3.33)	0.003	1.22 (0.77, 1.95)	0.393
Stable frail	17/5,194	3.29 (1.96, 5.54)	<0.001	Reference	
Frail to non-frail/pre-frail	11/3,589	2.63 (1.41, 4.91)	0.002	0.75 (0.31, 1.79)	0.485
**Osteoarthritis**					
Stable non-frail	567/96,090	Reference		Reference	
Non-frail to pre-frail/frail	387/44,967	1.35 (1.19, 1.54)	<0.001	1.34 (1.17, 1.52)	<0.001
Stable pre-frail	916/94,801	1.36 (1.22, 1.51)	<0.001	Reference	
Pre-frail to non-frail	151/21,950	1.12 (0.94, 1.34)	0.218	0.81 (0.68, 0.96)	0.014
Pre-frail to frail	118/8,195	1.82 (1.48, 2.23)	<0.001	1.37 (1.13, 1.66)	0.002
Stable frail	79/4,764	1.91 (1.50, 2.43)	<0.001	Reference	
Frail to non-frail/pre-frail	42/3,327	1.52 (1.11, 2.09)	0.009	0.83 (0.56, 1.22)	0.329
**Intervertebral disc degeneration**					
Stable non-frail	80/98,872	Reference		Reference	
Non-frail to pre-frail/frail	41/46,784	1.08 (0.74, 1.57)	0.708	1.10 (0.75, 1.62)	0.623
Stable pre-frail	143/99,289	1.64 (1.24, 2.17)	<0.001	Reference	
Pre-frail to non-frail	15/22,765	0.80 (0.46, 1.39)	0.419	0.49 (0.29, 0.84)	0.010
Pre-frail to frail	25/8,701	3.16 (2.00, 5.02)	<0.001	1.88 (1.22, 2.90)	0.005
Stable frail	17/5,218	3.19 (1.85, 5.51)	<0.001	Reference	
Frail to non-frail/pre-frail	7/3,603	2.00 (0.91, 4.37)	0.085	0.60 (0.21, 1.71)	0.300
**Degenerative bone and joint multimorbidity**					
Stable non-frail	55/99,107	Reference		Reference	
Non-frail to pre-frail/frail	39/46,919	1.49 (0.98, 2.25)	0.060	1.53 (1.00, 2.33)	0.050
Stable pre-frail	120/99,645	1.85 (1.34, 2.57)	<0.001	Reference	
Pre-frail to non-frail	21/22,838	1.58 (0.95, 2.62)	0.075	0.86 (0.54, 1.38)	0.541
Pre-frail to frail	19/8,771	3.27 (1.92, 5.59)	<0.001	1.68 (1.02, 2.75)	0.041
Stable frail	16/5,228	4.44 (2.48, 7.94)	<0.001	Reference	
Frail to non-frail/pre-frail	9/3,604	3.50 (1.71, 7.18)	<0.001	0.86 (0.33, 2.24)	0.727

In the cut-off value 1, non-frail was defined as FI ≤0.08, pre-frail was defined as 0.08< FI ≤0.25, frail was defined as FI >0.25. PYs represents the person years from first or second follow-up to DBJDs onset.

Abbreviations: PYs, person years; TDI, Townsend deprivation index; BMI, body mass index.

[a]HR and P were adjusted for age, sex, race, education, TDI, smoking status, drinking status, physical activity level and BMI.

Table S18. Association the rate of change in frailty index with incident degenerative bone and joint diseases and its multimorbidity excluding cases occurring within the first two years of follow-up.

Variables	Cases/PYs	HR(95%CI)[a]	P[a]
**Degenerative bone and joint diseases**			
T1 of △FI / year	920/95,892	Reference	
T2 of △FI / year	711/78,679	1.17 (1.06, 1.29)	0.003
T3 of △FI / year	1,173/96,353	1.35 (1.23, 1.48)	<0.001
P for trend test		<0.001	
△FI per 0.01-point/year increase		1.12 (1.08, 1.16)	<0.001
△FI per 0.05-point/year increase		1.75 (1.45, 2.10)	<0.001
**Osteoporosis**			
T1 of △FI / year	168/100,660	Reference	
T2 of △FI / year	129/82,044	1.30 (1.03, 1.65)	0.028
T3 of △FI / year	211/101,897	1.52 (1.23, 1.87)	<0.001
P for trend test		<0.001	
△FI per 0.01-point/year increase		1.12 (1.03, 1.22)	0.009
△FI per 0.05-point/year increase		1.76 (1.15, 2.70)	0.009
**Osteoarthritis**			
T1 of △FI / year	748/96,920	Reference	
T2 of △FI / year	583/79,384	1.16 (1.03, 1.29)	0.011
T3 of △FI / year	929/97,790	1.26 (1.14, 1.40)	<0.001
P for trend test		<0.001	
△FI per 0.01-point/year increase		1.09 (1.04, 1.13)	<0.001
△FI per 0.05-point/year increase		1.51 (1.23, 1.86)	<0.001
**Intervertebral disc degeneration**			
T1 of △FI / year	117/100,775	Reference	
T2 of △FI / year	76/82,245	1.07 (0.79, 1.44)	0.660
T3 of △FI / year	135/102,213	1.36 (1.05, 1.77)	0.019
P for trend test		0.018	
△FI per 0.01-point/year increase		1.23 (1.11, 1.36)	<0.001
△FI per 0.05-point/year increase		2.79 (1.66, 4.69)	<0.001
**Degenerative bone and joint multimorbidity**			
T1 of △FI / year	110/101,080	Reference	
T2 of △FI / year	71/82,403	1.19 (0.87, 1.62)	0.274
T3 of △FI / year	98/102,629	1.09 (0.82, 1.45)	0.560
P for trend test		0.555	
△FI per 0.01-point/year increase		1.04 (0.94, 1.17)	0.434
△FI per 0.05-point/year increase		1.25 (0.72, 2.16)	0.434

△FI was calculated by the FI at the final assessment minus the FI at baseline. T1 was the lower tertile, T2 was the middle tertile, and T3 was the upper tertile. PYs represents the person years from first or second follow-up to DBJDs onset.

Abbreviations: PYs, person years; TDI, Townsend deprivation index; BMI, body mass index.
[a]HR and P were adjusted for age, sex, race, education, TDI, smoking status, drinking status, physical activity level, BMI and baseline FI.

Table S19. Association of total frailty index with incident degenerative bone and joint diseases and its multimorbidity excluding cases occurring within the first two years of follow-up.

Variables	Cases/PYs	HR(95%CI)[a]	P[a]
**Degenerative bone and joint diseases**			
T1 of total FI	672/92,785	Reference	
T2 of total FI	893/88,927	1.27 (1.15, 1.40)	<0.001
T3 of total FI	1,239/89,212	1.59 (1.44, 1.75)	<0.001
P for trend test		<0.001	
Per 0.01-point increase		1.02 (1.01, 1.02)	<0.001
Per 0.05-point increase		1.08 (1.07, 1.10)	<0.001
**Osteoporosis**			
T1 of total FI	113/96,068	Reference	
T2 of total FI	170/93,273	1.50 (1.18, 1.91)	<0.001
T3 of total FI	225/95,261	1.98 (1.57, 2.49)	<0.001
P for trend test		<0.001	
Per 0.01-point increase		1.03 (1.02, 1.03)	<0.001
Per 0.05-point increase		1.13 (1.10, 1.17)	<0.001
**Osteoarthritis**			
T1 of total FI	540/93,580	Reference	
T2 of total FI	726/89,987	1.25 (1.12, 1.40)	<0.001
T3 of total FI	994/90,526	1.50 (1.35, 1.67)	<0.001
P for trend test		<0.001	
Per 0.01-point increase		1.01 (1.01, 1.02)	<0.001
Per 0.05-point increase		1.07 (1.05, 1.09)	<0.001
**Intervertebral disc degeneration**			
T1 of total FI	71/96,280	Reference	
T2 of total FI	87/93,470	1.21 (0.88, 1.66)	0.239
T3 of total FI	170/95,483	2.14 (1.60, 2.85)	<0.001
P for trend test		<0.001	
Per 0.01-point increase		1.03 (1.02, 1.04)	<0.001
Per 0.05-point increase		1.15 (1.11, 1.20)	<0.001
**Degenerative bone and joint multimorbidity**			
T1 of total FI	49/96,482	Reference	
T2 of total FI	84/93,804	1.60 (1.12, 2.28)	0.010
T3 of total FI	146/95,825	2.46 (1.76, 3.43)	<0.001
P for trend test		<0.001	
Per 0.01-point increase		1.03 (1.03, 1.04)	<0.001
Per 0.05-point increase		1.18 (1.13, 1.23)	<0.001

Total FI was calculated by the FI at baseline plus the FI at the final assessment. T1 was the lower tertile, T2 was the middle tertile, and T3 was the upper tertile. PYs represents the person years from first or second follow-up to DBJDs onset.

Abbreviations: PYs, person years; TDI, Townsend deprivation index; BMI, body mass index.
[a]HR and P were adjusted for age, sex, race, education, TDI, smoking status, drinking status, physical activity level and BMI.
